# scLTF: A self-training clustering framework for large-scale single-cell RNA-Seq data

**DOI:** 10.1371/journal.pone.0359153

**Published:** 2026-09-24

**Authors:** Hongyi Yuan, Chunyan Wang, Qiucheng Sun, Mingyu Sun, Mujie Fan

**Affiliations:** School of Artificial Intelligence, Changchun Normal University, Changchun, China; State University of New York at Oswego, UNITED STATES OF AMERICA

## Abstract

Single-cell RNA sequencing (scRNA-seq) technology has rapidly advanced in recent years, driving significant breakthroughs in developmental biology, cancer research, immunology, and other related fields. However, existing clustering methods still face performance bottlenecks when handling large-scale scRNA-seq data. To address this challenge, we propose a self-training clustering method for large-scale single-cell RNA-seq data, termed scLTF (Single-cell Louvain-Transformer Framework for Large-scale Clustering). This method first generates preliminary clusters using a fast Louvain algorithm and then selects key cells based on a custom “Cluster Representativeness Index”. Subsequently, a Transformer model is employed for iterative representation learning and optimization to optimize the final clustering results. Experiments on multiple real-world datasets demonstrate that scLTF achieves overall superior or comparable performance in clustering accuracy compared with several state-of-the-art deep clustering methods, with evaluation metrics (ARI, NMI, and ACC) improving on average by approximately 3.9%–24.1%; meanwhile, its runtime is only about 6.7%–43.9% of existing deep learning-based methods. scLTF integrates the advantages of traditional graph-based clustering and deep learning models, achieving both high clustering performance and computational efficiency, thus providing a practical and reliable tool for biomedical research and cellular analysis.

## Introduction

Single-cell RNA sequencing (scRNA-seq) technology has rapidly advanced in recent years [1], becoming an essential tool for studying cellular heterogeneity [2,3]. Compared with traditional bulk sequencing, scRNA-seq captures gene expression differences at the single-cell level, enabling the identification of rare cell subpopulations and the elucidation of complex developmental processes and disease mechanisms [4,5]. This technology has been widely applied in fields such as immunology, oncology, and developmental biology, and has facilitated the emergence of single-cell multi-omics research [6–8]. In addition, computational and systems biology approaches offer complementary insights into complex biological systems [9]. However, as data scale continues to increase, its high dimensionality and sparsity become more pronounced. In high-dimensional space, traditional distance metrics (e.g., Euclidean distance) often suffer from the “distance concentration phenomenon,” making it difficult to effectively distinguish similarities between different cells and thereby weakening clustering performance based on distance or graph structures. In addition, a large number of “dropout” zero values disrupt the continuity of local neighborhood structures, distorting the true topological relationships among cells and further increasing clustering difficulty [10]. Meanwhile, technical noise and batch effects introduce additional non-biological variation signals, causing clustering results to deviate from the true cell-type structure [11]. Therefore, developing clustering methods that exhibit computational efficiency, robustness, and biological interpretability has become a key challenge in current scRNA-seq data analysis [12,13].

Early clustering methods mainly include distance- or model-based algorithms, such as K-means, hierarchical clustering, and Gaussian mixture models (GMMs). K-means [14] partitions cells by minimizing within-cluster squared distances, but it is sensitive to noise and the choice of initial cluster centers in high-dimensional and sparse data. Hierarchical clustering [15] can reveal hierarchical relationships among cells, yet its results are easily degraded by noise when applied to large-scale or complex datasets. GMMs [16] perform clustering by assuming an underlying data distribution, but their performance depends heavily on parameter selection and they have limited scalability. With the increasing scale and complexity of single-cell datasets, graph-based clustering methods have also been widely adopted in scRNA-seq analysis. The Louvain algorithm [17] identifies cell populations by maximizing modularity; however, its performance is highly dependent on the quality of the constructed k-nearest neighbor (k-NN) graph and the choice of resolution parameters, leading to substantial variability under different graph structures. In sparse graph settings, modularity optimization suffers from the resolution limit problem, which may result in over-partitioning or unstable community structures. The Leiden algorithm [18] improves partition consistency by refining local optimization strategies; however, in complex large-scale datasets, it may still exhibit insufficient fine-grained partitioning or local structural deviations.. In addition, density-based methods such as DBSCAN [19] can identify clusters of arbitrary shapes and automatically handle noise, but they are sensitive to data sparsity and require carefully tuned parameters in high-dimensional settings. Spectral clustering [20], which constructs a similarity matrix and performs Laplacian spectral dimensionality reduction, can detect non-convex clusters; however, its performance degrades in high-dimensional and sparse settings because similarity estimation becomes less reliable. Fuzzy clustering methods, such as Fuzzy C-Means (FCM) [21], allow cells to belong to multiple clusters and are suitable for capturing continuous cellular states, but they are prone to local optima in noisy and sparse datasets. To enhance clustering robustness, ensemble methods have been proposed. For example, SC3 [22] constructs a consensus matrix from multiple clustering algorithms to obtain more stable results; however, the multi-round clustering process and matrix computations introduce substantial computational complexity, limiting its applicability and accuracy on large-scale datasets. Overall, although these methods are effective in different scenarios, their common limitations can be summarized into three categories: (1) high sensitivity to graph construction strategies or parameter settings, leading to limited result stability; (2) distorted similarity measurements in high-dimensional sparse spaces, making it difficult to accurately recover the true topological structure of cells; and (3) a trade-off between clustering accuracy and computational efficiency, which becomes particularly pronounced in large-scale datasets. Furthermore, these issues collectively make it challenging for existing methods to simultaneously ensure stability, accuracy, and efficiency under the combined challenges of high dimensionality, sparsity, large-scale samples, and noise interference.

With the increasing use of deep learning in large-scale data processing and nonlinear feature extraction, it has also been widely applied to scRNA-seq clustering analysis. Autoencoders (AEs) and their variants, such as DCA [23] and SAUCIE [24], extract latent cellular features through denoising and dimensionality reduction, thereby alleviating challenges arising from high dimensionality and data sparsity. By learning cellular phenotypic representations in a low-dimensional latent space, these methods can capture complex nonlinear relationships and partially mitigate technical noise and dropout effects [25]. Building upon this idea, graph neural network (GNN)–based methods, such as scGNN [26], construct cell–cell graphs for feature aggregation and use graph convolution to capture both local and global structural information, thereby improving clustering accuracy and interpretability. In contrast, scGGAN [27] employs a graph-based generative adversarial network to impute missing values in scRNA-seq data, further enhancing data quality and clustering performance. On the other hand, methods based on variational autoencoders, such as cellVGAE [28], model cellular relationships via graph-based encoding and decoding, improving clustering performance and stability; however, they incur high computational cost, particularly when applied to large-scale single-cell datasets. In addition, other deep-learning-based clustering strategies have received considerable attention. For example, the scVI framework [29] employs a generative model to perform probabilistic modeling of single-cell expression data; and the recently proposed scTrans [30] leverages a Transformer architecture with sparse attention mechanisms to process scRNA-seq data. Although deep learning methods have demonstrated significant advantages in capturing nonlinear features and improving clustering accuracy, their common bottlenecks have also become increasingly apparent. Training deep neural networks requires substantial computational resources, leading to long runtimes on large-scale datasets. In addition, issues such as gradient instability and memory overflow frequently arise in practical applications, limiting their scalability to even larger datasets [25].

In recent years, self-training [31] has been increasingly introduced into scRNA-seq data analysis to alleviate challenges arising from limited labeled data as well as high dimensionality and data sparsity. It is important to emphasize that, in such methods, the “supervisory signals” are derived from pseudo-labels generated by the model itself or from the initial clustering process, rather than external human annotations, forming a pseudo-label–based self-training learning paradigm [32]. These methods typically first generate initial clustering results or pseudo-labels using unsupervised models, and then iteratively refine them using deep learning models to improve feature representation and clustering performance. In the field of single-cell analysis, scDeepCluster [12] integrates deep embedding learning with a pseudo-label iterative mechanism, achieving joint optimization of representation learning and clustering through continuously updated cluster assignments. DFCN [33] constructs a unified framework for feature learning and clustering assignment, iteratively updating pseudo-labels during training to improve clustering quality. scDCC [34] employs deep representation learning combined with a self-training mechanism to enhance cell-type discriminability through continuous pseudo-label refinement. Related studies have shown that such structure-guided supervisory strategies can effectively exploit the intrinsic structure of data and improve clustering accuracy and robustness without relying on manual annotations. However, existing self-training methods still face two main challenges: first, the quality of initial pseudo-labels significantly affects subsequent model training, and erroneous labels may be amplified during iterative optimization; second, complex deep models often incur high computational costs, limiting their scalability in large-scale scRNA-seq data analysis. Therefore, how to select high-confidence samples from initial clustering results and design lightweight yet efficient deep models for clustering refinement remains an important direction in self-training-based clustering research.

To address the aforementioned challenges, we propose a self-training clustering method that integrates a fast Louvain algorithm with a Transformer model, termed scLTF. The method first applies an optimized fast Louvain algorithm (FLA) to generate preliminary clusters of scRNA-seq data, and then selects key cell samples based on a custom Cluster Representativeness Index (CRI) to enhance clustering robustness and accuracy. Subsequently, a Transformer model is introduced as a self-training optimization module. It performs representation learning on the CRI-selected key cells and uses pseudo-labels generated from the initial clustering results as supervisory signals. In this way, it learns discriminative cellular representations and further refines the clustering structure to obtain the final clustering results. scLTF can handle high-dimensional, sparse, and noisy single-cell data, and demonstrates good computational efficiency on large-scale datasets. Experimental results on multiple public datasets indicate that the proposed method shows certain advantages in clustering performance and applicability, providing a solution for large-scale scRNA-seq data analysis that balances efficiency, scalability, and biological interpretability.

The main contributions of this study are as follows:

(1) We propose a self-training clustering framework for large-scale scRNA-seq data (scLTF), which leverages fast graph-based clustering to generate reliable preliminary clustering results, and incorporates a Transformer for representation learning. A pseudo-label mechanism is further employed to iteratively refine clustering results;
(2) In the initial clustering stage, an optimized fast Louvain algorithm (FLA) is introduced. By combining representative anchor points with a sparse neighborhood graph strategy, computational overhead is reduced, enabling efficient and stable initial clustering for large-scale scRNA-seq data;
(3) A Cluster Representativeness Index (CRI) is designed to select representative cells from the initial clustering results, reducing the influence of low-quality labels on model training and improving the stability and robustness of the self-training process.
(4) Experimental results on multiple public scRNA-seq datasets show that the proposed method outperforms multiple representative methods in terms of clustering accuracy, runtime efficiency, and scalability, demonstrating potential for practical applications.

## Methods

### Overview of scLTF

**Fig 1:**
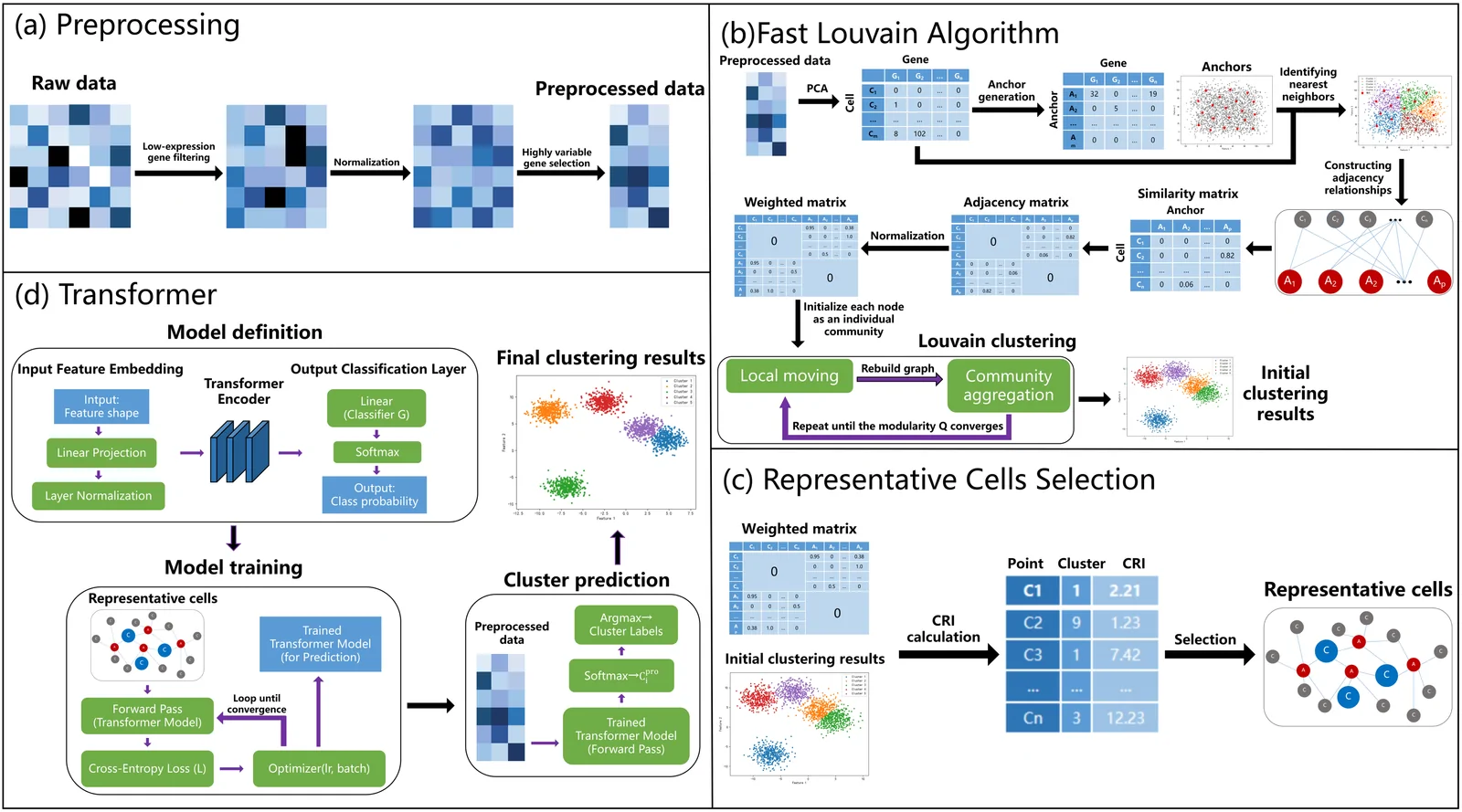
Overall workflow of scLTF. (a) Data preprocessing; (b) Initial clustering using the fast Louvain Algorithm; (c) Representative cell selection; (d) Transformer-based clustering.

As illustrated in Fig 1, scLTF consists of four major modules: data preprocessing, initial clustering using an improved fast Louvain algorithm, representative cell selection, and final clustering using a Transformer model. In the first module, the raw scRNA-seq data is preprocessed. In the second module, the preprocessed data are clustered using the fast Louvain algorithm (FLA) to generate preliminary clusters. In the third module, the custom-defined Clustering Representativeness Index (CRI) is employed to evaluate and select representative cells from each preliminary cluster. In the fourth module, the cluster labels corresponding to the selected representative cells are used as pseudo-labels to train the Transformer model. Finally, the trained model is applied to the entire dataset to obtain the final clustering results.

### Data preprocessing

After obtaining single-cell RNA sequencing (scRNA-seq) data, we first performed data preprocessing, including lowly expressed gene filtering, normalization, and highly variable gene selection. Specifically, genes expressed in fewer than five cells were removed by calculating the expression prevalence of each gene across all cells, thereby eliminating low-expression noise and reducing redundant features. Next, log-normalization was applied to the filtered gene expression matrix, formulated as follows:


$$\begin{array}{c} {x{\;}^{\prime }}_{ij}=log(\text{1}+x_{ij}) \end{array}$$ (1)

Where ${\text{x}}_{\text{ij}}$ denotes the raw expression value of gene i in cell j, and ${{\text{x}}^{\prime }}_{\text{ij}}$ represents the log-normalized expression value. Subsequently, the variance of each gene across all cells was calculated, and the top 2,000 genes with the highest variance were selected as highly variable genes (HVGs).

### Preliminary clustering with the fast Louvain algorithm

To address the issue of high computational cost of the traditional Louvain algorithm when processing large-scale single-cell RNA sequencing data, this study designs an improved fast Louvain algorithm (FLA) to improve computational efficiency while enhancing the stability and scalability of clustering results. After data preprocessing, the algorithm is used for initial clustering of single-cell data. The core idea of FLA is to introduce an anchor selection strategy and a sparse matrix construction strategy before clustering, in order to reduce redundant computations and lower the overall computational cost. The specific steps are as follows:

First, principal component analysis (PCA) is applied to the gene expression matrix [35], and the top 50 principal components are retained as low-dimensional feature representations. This setting is kept consistent across all datasets, thereby projecting the high-dimensional data into a lower-dimensional space and reducing the dimensionality burden for subsequent computations.

Subsequently, to avoid the high computational cost associated with constructing a large-scale dense matrix directly, we generate a set of representative anchors to approximate the underlying geometric structure of the data. Specifically, p_0_ candidate cells are randomly selected from all N cells and clustered into p clusters using K-means [14], where p_0_ = min(10p, N). The centroids of these clusters serve as the final anchors r_1_, r_2_, …, r_p_.

After generating the anchors, k local neighboring anchors are selected for each cell based on Euclidean distance to approximate its neighborhood structure. This strategy reduces the computational burden via approximate calculations and constructs a sparse cell–anchor adjacency, thereby further reducing the data scale. The specific steps are as follows:

- The p anchors are further clustered into M clusters using the K-means algorithm, where M = √p.
- Each cell X_i_ is assigned to the nearest cluster based on its distance to the cluster centroids, and the closest anchor r_i_ within that cluster is selected.
- For the selected anchor r_i_, a K-nearest neighbors (KNN) algorithm [36] is applied within its cluster to select k_0_ nearest anchors as a candidate set, where k_0_ = 5k.
- From these k_0_ candidate anchors, the k nearest anchors to cell X_i_ are selected as its local neighboring anchors.

After selecting the k local neighboring anchors, a locally scaled Gaussian kernel [36] is applied to convert the distances between cells and anchors into similarity measures, to generate a sparse similarity matrix B, where rows correspond to cells and columns correspond to anchors. For each row, only the similarity values with the local neighboring anchors are retained, and all other elements are set to zero, thereby reducing computational cost while preserving the primary relational structure. The locally scaled Gaussian kernel is defined as follows:


$$\begin{array}{c} B_{ij}=exp\left(-\frac{{d\left(X_{i},\;r_{j}\right)}^{\text{2}}}{\text{2}\sigma^{\text{2}}}\right) \end{array}$$ (2)

Here, i= 1, 2, …, N indexes all cells,  j= 1, 2, …, p denotes all anchors, $B_{ij}$ denotes the similarity between cell ${\text{X}}_{\text{i}}$ and anchor ${\text{r}}_{\text{j}}$, $d\left(X_{i},\;r_{j}\right)$ is the Euclidean distance, and σ is a Gaussian kernel bandwidth parameter estimated adaptively as the mean distance between cell i and its k local neighboring anchors. Subsequently, based on the sparse similarity matrix B, a sparse adjacency matrix W with dimensions (N + p) × (N + p) is constructed as follows:


$$\begin{array}{c} W=[\begin{array}{cc} \text{0} & B\\ B^{T} & \text{0} \end{array}] \end{array}$$ (3)

The upper-left block is an N × N zero matrix (representing no direct connections between cells), and the bottom-right block is a p × p zero matrix (representing no direct connections between anchors). As a result, only cross-layer connections between cells and anchors are preserved, forming a bipartite graph structure. Subsequently, for numerical convenience and to improve implementation stability, the edge weights are linearly rescaled using min–max normalization to obtain the normalized weight matrix A. This operation serves as a numerical preprocessing step to unify the scale of edge weights without altering the graph topology or the relative magnitudes of edges.

Subsequently, the matrix A is fed into the Louvain algorithm [17] for community detection. Since the matrix is sparse, the Louvain algorithm typically converges in relatively few iterations in practice, thereby improving clustering efficiency. The algorithm performs community detection by maximizing the modularity Q, which is defined as follows:


$$\begin{array}{c} Q=\frac{\text{1}}{\text{2}m}\sum_{i,\;j}\left(A_{ij}-\frac{k_{i}k_{j}}{\text{2}m}\right)\delta \left(c_{i},\;c_{j}\right) \end{array}$$ (4)

Here, $k_{i}$ and $k_{j}$ are the degrees of nodes i and j, respectively; m is the sum of all edge weights; $c_{i}$ and $c_{j}$ denote the community labels of the nodes; and $\delta \left(c_{i},\;c_{j}\right)$ is the Kronecker delta function, which taking the value if $c_{i}$=$c_{j}$, and 0 otherwise.

It should be noted that only the community labels assigned to cells are retained as the final clustering results, while the anchors are not included in subsequent analyses. The consistency between cell labels and anchor labels during community detection is further quantitatively evaluated in the experimental section.

The parameters p, k and p_0_ are evaluated in the experiments through sensitivity analysis to assess the impact of different settings on model performance. Other parameters are mainly used for auxiliary structural construction and have limited influence on the final graph structure. Empirically, no significant performance variation is observed under different settings; therefore, unified parameter values are adopted across all datasets to ensure reproducibility and fairness.

To analyze the computational efficiency of FLA, we evaluate the time complexity of its main steps. The complexity of the anchor generation and cell–anchor distance computation stage is O(N·p), while the local neighborhood selection step has a complexity of O(N). The constructed sparse bipartite graph contains O(N·k) edges; thus, the complexity of the Louvain algorithm is O(E) = O(N·k). Therefore, the overall time complexity of FLA is O(N·p + N·k). Since typically p ≫ k, it can be approximated as O(N·p).

In comparison, the standard KNN-based Louvain method first constructs a K-nearest neighbor graph over all samples, followed by community detection on the resulting sparse graph with O(N·k) edges. Therefore, the overall time complexity of the standard “KNN + Louvain” pipeline can be expressed as O(N^2^ + N·k)≈O(N^2^).

From a theoretical perspective, FLA reduces the graph construction time complexity from O(N^2^) to O(N·p) by introducing a cell–anchor bipartite sparse graph, thereby improving computational efficiency on large-scale datasets.

In addition, in terms of space complexity, FLA requires temporary storage of O(N·p) and O(p^2^) for cell–anchor and anchor–anchor distance computations, respectively, while the storage complexity of the sparse cell–anchor bipartite graph is O(N·k). Therefore, the overall space complexity of FLA is O(N·p + p^2^ + N·k). In the actual implementation, a sparse adjacency representation in CSR format is adopted to retain only local connectivity, thereby reducing the storage overhead compared with fully connected or dense similarity matrix-based methods.

It should be noted that, in practical applications, the actual runtime and memory overhead of the algorithm are also influenced by factors such as the system environment and data characteristics. Therefore, discrepancies may exist between theoretical complexity analysis and observed empirical performance.

### Representative cell selection

We propose a Clustering Representativeness Index (CRI) to evaluate and select representative cells within each cluster. Unlike traditional measures such as modularity or cluster centrality, CRI is designed to quantify both the consistency of each cell with the main structural pattern of its cluster and its representativeness in the expression feature space, thereby identifying cells that can better represent the overall characteristics of the cluster and reducing the number of samples involved in subsequent Transformer training, which helps improve computational efficiency. The specific steps are as follows:

Based on the preliminary clustering results generated by FLA and the weight matrix A, the CRI value for each cell is computed within its corresponding cluster. It should be noted that matrix A is the normalized weight matrix of the cell–anchor bipartite graph, which does not contain any cell–cell edges and only describes the connections between cells and anchors. A higher CRI value indicates that the cell has stronger connections with multiple anchors within its cluster, exhibits greater consistency with the main structural pattern of the cluster, and has stronger representativeness in the expression feature space. The CRI is calculated as follows:


$$\begin{array}{c} {CRI}_{i}=\sum_{j\in C_{i}}A_{ij} \end{array}$$ (5)

Here, ${CRI}_{i}$ denotes the clustering representativeness of node i within its cluster, $C_{i}$ denotes the set of anchors contained in the initial cluster to which cell i belongs, and $A_{ij}$ represents the element in the sparse weight matrix A, indicating the normalized connection weight between cell i and anchor j within the cluster. In this bipartite graph, anchors serve as shared intermediates that map similarity in the expression feature space into cell–anchor connections. Multiple anchors within the same preliminary cluster jointly characterize the main structural pattern of the cluster. Therefore, CRI reflects the degree of consistency between a cell and the main structural pattern of its cluster by aggregating the connection weights between the cell and anchors within the same cluster, and further characterizes its representativeness in the expression feature space.

After computing the CRI values for all cells, a certain proportion of cells with the highest CRI values is selected from each cluster as representative cells, with at least one representative cell selected per cluster to avoid empty selection in small clusters. This proportion can be adjusted empirically across different datasets, and its influence on model performance is evaluated through sensitivity analysis in the experiments. The set of representative cell indices is defined as follows:


$$\begin{array}{c} R={⋃}_{k}{RepCells}_{k} \end{array}$$ (6)

Here, ${\text{RepCells}}_{\text{k}}$ denotes the set of representative cell indices selected from the k-th initial cluster, and R denotes the set of all representative cell indices.

These representative cells can better capture the core characteristics of their respective clusters while reducing the amount of data used for subsequent model training. This may help reduce computational costs and facilitate more effective clustering refinement.

### Final clustering with the Transformer model

After selecting the representative cells, we employ a Transformer-based deep learning model [37] to perform nonlinear representation learning on single-cell gene expression features, thereby enhancing the discriminative power and separability of the learned representations. The proposed method adopts a self-training framework based on clustering results, where the labels of representative cells are generated from the initial FLA clustering results and used as pseudo-labels to guide model training. The trained model is then applied to all cells to obtain the final clustering results.

Because the input consists of unordered gene expression feature vectors, no explicit positional encoding is introduced. Instead, the Transformer is employed to capture nonlinear dependencies among feature dimensions. The Transformer model consists of an input feature embedding layer, multi-head self-attention encoder layers, and an output classification layer. Each encoder layer is composed of a multi-head self-attention mechanism (Multi-Head Self-Attention) and a feed-forward neural network (FFN), together with residual connections and layer normalization. First, the input gene expression matrix X is projected into the model’s hidden dimension and subjected to layer normalization, as expressed by the following equation:


$$\begin{array}{c} H_{\text{0}}=LayerNorm(XW^{input}+b^{input}) \end{array}$$ (7)

Here, $W^{input}$ denotes the weight matrix, and $b^{input}$ denotes the bias term. LayerNorm denotes layer normalization, which stabilizes training and improves model convergence. $H_{\text{0}}$ is the final feature representation input to the encoder. To mitigate the risk of overfitting caused by the limited number of representative cells, multiple regularization strategies are incorporated during model training, including the Dropout mechanism, L2 weight decay, and an Early Stopping strategy based on validation loss. These techniques are used to reduce overfitting to pseudo-labels and improve the stability and robustness of the training process.

The feature matrix is subsequently processed by the Transformer encoder to learn higher-order nonlinear mappings of cell representations in the feature space. The encoded representation is then projected onto the output classes through a linear classification layer, producing the predicted probability distribution for each cell:


$$\begin{array}{c} Y=Softmax(ZW^{out}+b^{out}) \end{array}$$ (8)

Here, Z denotes the output of the final encoder layer, and $W^{out}$ and $b^{out}$ are the weight matrices and bias terms, respectively.

The model uses representative cells as pseudo-label–supervised samples for training, and is optimized by minimizing the cross-entropy loss between the predicted outputs and the pseudo-labels, as formulated below:


$$\begin{array}{c} L=-\sum_{\text{i}\in \text{R}}\sum_{\text{c}=\text{1}}^{\text{n}}C_{i}^{(c)}log(Y_{i}^{(c)}) \end{array}$$ (9)

Here, $C_{i}$ denotes the pseudo-label assigned to cell i by the preliminary clustering result, $Y_{i}^{(c)}$ is the model–predicted probability that a cell belongs to class c, and n is the number of clusters obtained from the preliminary clustering, and R denotes the index set of representative cells obtained in the representative cell selection stage.

Notably, the Transformer model is not simply a label propagation process; instead, it performs end-to-end representation learning to jointly optimize feature representations and classification decision boundaries. With the number of clusters and class definitions determined by the initial FLA clustering results and kept fixed, the Transformer predicts the class of each cell based on the learned feature representations and classification decision boundaries, allowing some cells to be reassigned to other existing classes. Therefore, the refinement in this study refers to improving the assignment of cells to existing clusters within a fixed class space, rather than generating new clustering structures, changing the number of clusters, or redefining the classes.

To ensure reproducibility and stability of the training process, key hyperparameters of the Transformer model are carefully set and tuned. The specific parameters, along with their default values or tuning ranges, are listed in Table 1.

**Table 1:** Hyperparameter settings of the Transformer model.

Parameter	Default Value/ Range	Tuning Strategy
Learning rate	[0.1, 0.01, 0.001, 0.0001]	Grid search
Batch size	[16, 32, 64, 128]	Grid search
Number of attention heads	4–8	Empirically set
Number of encoder layers	2–3	Empirically set
Hidden dimension	256	Empirically set
Dropout rate	0.3	Empirically set
Early stopping patience	10	Monitored on validation set

Training parameters are iteratively updated using an optimizer during the training process, while strategies such as learning rate scheduling and batch size adjustment are adopted to improve training stability and generalization ability. The proposed method is trained under a pseudo-label supervision framework, where the labels of representative cells are derived from the initial clustering results rather than manual annotations..

After training, the full gene expression matrix is input into the Transformer encoder to generate feature representations for all cells, and the same classification layer is used to obtain the final class probability distributions. For each cell, the predicted label is determined by the class with the highest probability, as formulated below:


$$\begin{array}{c} {Cluster}_{i}=\underset{c}{argmax}Y^{full}[i,\;c] \end{array}$$ (10)

Here, argmax denotes the index of the maximum value in the input vector, $Y^{full}$ represents the softmax-normalized predicted probability distribution of each cell over n classes, where c = 1, 2, …, n denotes the class index, and ${Cluster}_{i}$ represents the final cluster label of cell i.

Through the above process, the Transformer model learns nonlinear feature representations and classification decision boundaries under pseudo-label supervision and performs final class assignment for all cells based on the predicted probabilities, thereby completing the final clustering of the entire dataset.

### Clustering evaluation metrics

To comprehensively evaluate the performance of clustering algorithms, this study employs three widely used metrics: Adjusted Rand Index (ARI) [38], Normalized Mutual Information (NMI) [39], and Clustering Accuracy (ACC) [40]. By jointly employing the three metrics, the clustering quality can be evaluated from multiple perspectives, including consistency, mutual information relevance, and accuracy. Comparing different algorithms across these metrics enables a rigorous assessment of the stability and superiority of the proposed method on various datasets.

## Results

### Dataset description

In this study, we selected eight publicly available scRNA-seq datasets to evaluate and validate the proposed method. These datasets span a wide range of biological contexts, covering both human and mouse samples and encompassing various cell types such as cardiac progenitor cells, induced pluripotent stem cells (iPSCs), liver endothelial cells, peripheral blood mononuclear cells (PBMCs), splenocytes, and specific tumor cell lines. The datasets vary significantly in scale, with cell numbers ranging from tens of thousands to over 300,000, and gene counts ranging from approximately 15,000–37,000, reflecting substantial diversity and complexity. This variability not only reflects the biological characteristics of different tissues and cell populations but also provides a systematic foundation for evaluating single-cell analysis methods in terms of their ability to handle large-scale data, cope with cell-type heterogeneity, and generalize across species. All datasets were obtained from public repositories, such as NCBI, 10x Genomics, and Figshare, with detailed information provided in Table 2.

**Table 2:** Overview of the real-world scRNA-seq datasets used in this study.

Dataset	Cells	Genes	Clusters	Species	Cell type	Origin
GSE220195	11,754	14,949	8	Mouse	Cardiac progenitors/ iPSCs	NCBI
GSE198868	23,736	22,507	26	Mouse	Liver endothelial cells	NCBI
20k Human PBMCs, 3’ HT v3.1, Chromium X	23,837	36,601	18	Human	PBMCs	10x Genomics
GSE223414	45,460	25,639	12	Human	Cardiac progenitors/ iPSCs	NCBI
Aggregate of 8 Chromium Connect channels and 8 manual channels	66,457	33,538	26	Human	PBMCs	10x Genomics
128k Mouse Splenocytes, Multiplexed Samples, 16 Probe Barcodes	113,001	19,059	49	Mouse	Splenocytes	10x Genomics
128k Jurkat Cells, Multiplexed Samples, 16 Probe Barcodes	145,628	18,082	18	Human	Jurkat cells	10x Genomics
MCA DGE Data	333,778	34,947	104	Mouse	Various mouse cells	Figshare

To ensure comparability across different datasets, all datasets were processed using a unified preprocessing pipeline as described in the Data Preprocessing section of the Methods, including low-expression gene filtering, normalization, and highly variable gene selection. This study aims to independently evaluate the clustering performance and robustness of the proposed method within each dataset. Each dataset is treated as an independent analytical unit and processed separately, without performing cross-dataset joint modeling or data integration. Therefore, no explicit batch effect correction was introduced in the experimental setup of this study.

The ground truth labels used for clustering performance evaluation were obtained from cell type annotations provided in the corresponding publicly available datasets and were used as the reference standard for computing evaluation metrics such as ARI, NMI, and ACC.

### Description of clustering algorithms for comparison

The scLTF algorithm incorporates FLA, using its clustering results as an initial foundation and further refining the clustering structure. Since this two-stage strategy inevitably increases computational time while improving clustering accuracy, a range of traditional clustering algorithms and deep learning-based clustering methods were selected in the comparative experiments to evaluate the performance of FLA and scLTF in terms of both clustering accuracy and computational efficiency.

To comprehensively evaluate the applicability of FLA across datasets with varying scales and complexities, we selected several representative traditional clustering methods for comparison. Seurat [6] and Scanpy [10] are among the most widely used toolkits in the field of single-cell analysis, both of which employ adjacency graph-based community detection strategies for clustering. Leiden [18], as a graph-based community detection algorithm, effectively identifies structural relationships among cells by optimizing the community partition. K-means [14], as a classical clustering method, is renowned for its efficiency and simplicity but shows limited performance when handling non-spherical clusters.

In addition, to validate the advantages of the scLTF algorithm in improving clustering accuracy and efficiency, we selected several deep learning–based clustering methods as benchmarks. scVI [29] is a generative representation learning model based on variational autoencoders (VAEs). It performs latent space modeling of scRNA-seq data to obtain low-dimensional representations that capture nonlinear relationships among cells. In this study, scVI is combined with the Leiden community detection algorithm for clustering. Specifically, scVI is first used to learn latent representations, after which a cell–cell adjacency graph is constructed in the latent space, and community detection is performed using the Leiden algorithm to obtain the final clustering results. scTPC [41] constructs a topology-preserving cellular representation space and integrates graph-based clustering strategies to jointly model both local and global structures of cells. It demonstrates strong clustering performance in datasets with complex cellular heterogeneity.scMAE [42] integrates a masking mechanism with an autoencoder architecture, which not only alleviates the sparsity and dropout issues inherent in scRNA-seq data but also extracts high-quality low-dimensional representations to enhance clustering discriminability. scDeepCluster [12] jointly optimizes the autoencoder and clustering objectives, demonstrating strong capability in capturing complex nonlinear structures and revealing potential cell subpopulations, albeit at a higher computational cost.

Through this two-tiered comparison framework, we are able to comprehensively evaluate the overall performance of the FLA and scLTF algorithms in terms of both accuracy and computational efficiency. The hardware and software environments and specific implementation settings used in the experiments are detailed in S1 File.

### Clustering performance evaluation

**Fig 2:**
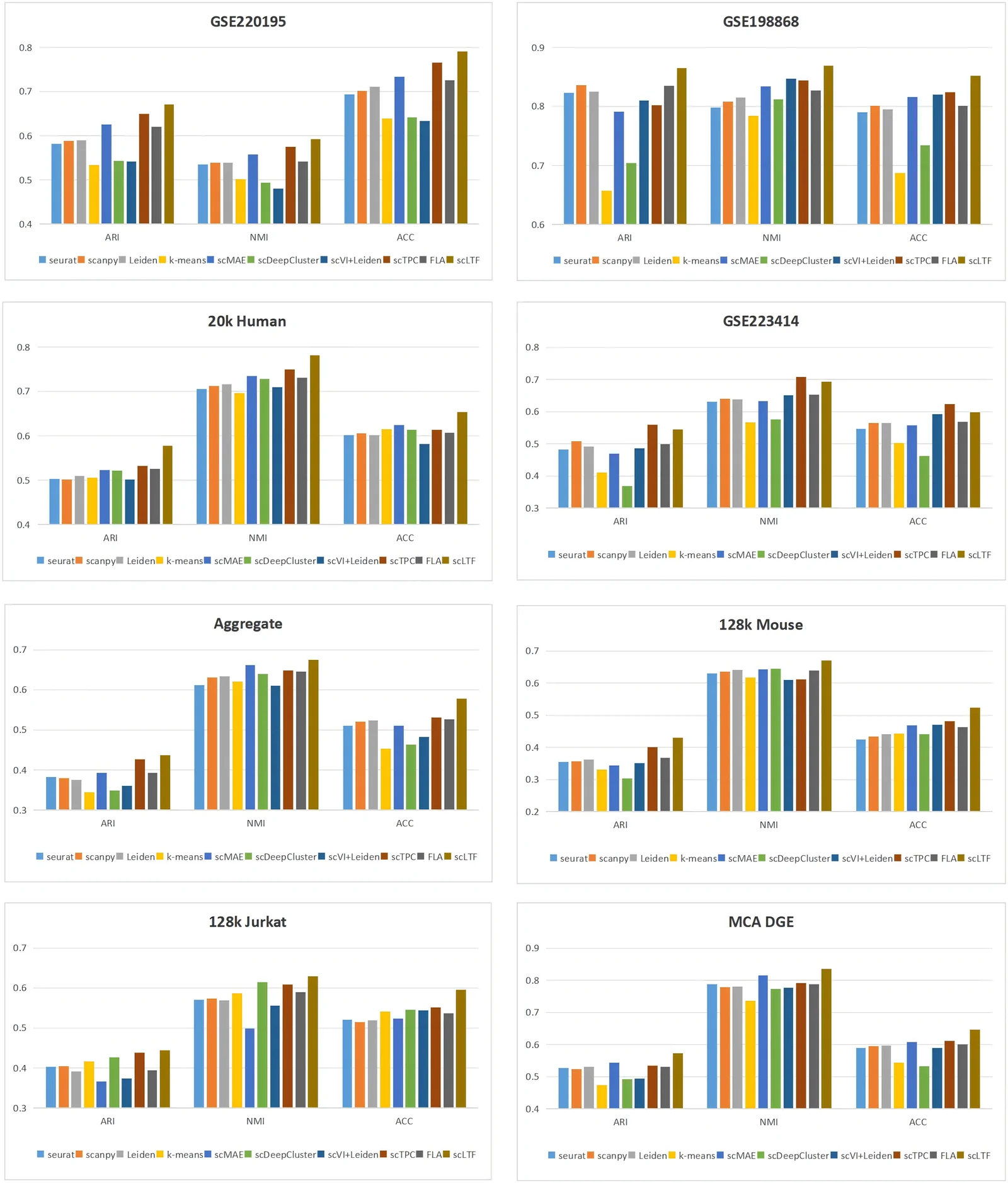
Comparison of clustering evaluation metrics for FLA, scLTF, and eight other clustering algorithms across eight datasets.

**Fig 3:**
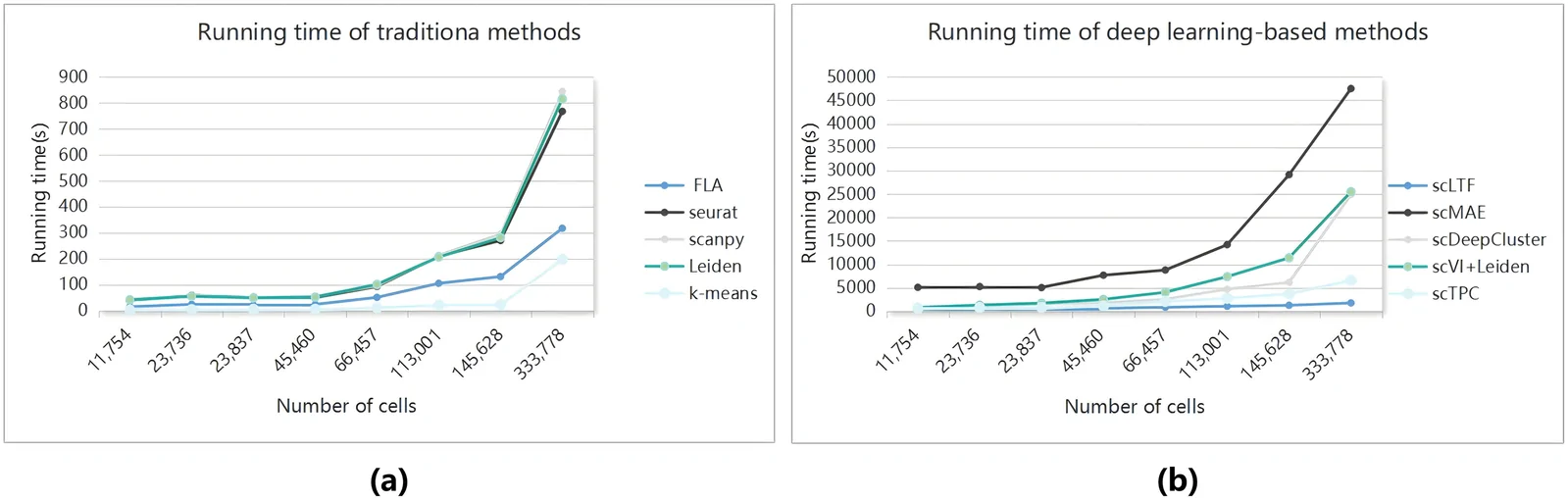
Comparison of clustering runtimes. (a) Runtime comparison of FLA and four other traditional clustering algorithms across eight datasets; (b) Runtime comparison of scLTF and four other deep learning-based clustering algorithms across eight datasets.

To comprehensively assess the performance of different clustering algorithms, this study conducted a systematic comparison of FLA, scLTF, and several mainstream clustering methods based on eight real single-cell transcriptomic datasets. Evaluation metrics included ARI, NMI, and ACC to quantify the clustering accuracy of each algorithm on each dataset. The clustering performance of the algorithms across the eight datasets is shown in Fig 2. In addition, we recorded the runtime of each algorithm on different datasets to evaluate computational efficiency, with results presented in Fig 3. A comprehensive assessment of the overall performance of all methods was conducted by jointly considering clustering accuracy and computational efficiency.

First, from the perspective of clustering accuracy, we evaluate the performance of FLA against traditional methods. Specifically, in terms of the ARI metric, FLA outperforms Seurat in 7 out of 8 datasets, Scanpy in 5 out of 8 datasets, Leiden in 7 out of 8 datasets, and K-means in 7 out of 8 datasets. For the NMI metric, FLA achieves better performance than Seurat in 7 out of 8 datasets, Leiden in 7 out of 8 datasets, and consistently outperforms both Scanpy and K-means across all 8 datasets. Regarding the ACC metric, FLA outperforms Seurat and Leiden on all 8 datasets, Scanpy on 7 out of 8 datasets, and K-means on 6 out of 8 datasets. In terms of average improvement, FLA achieves gains of 2.5%, 2.8%, and 3.5% over Seurat in terms of ARI, NMI, and ACC, respectively. Compared with Scanpy, the improvements are 1.7%, 1.8%, and 2.2%, respectively. Compared with Leiden, FLA achieves improvements of 2.2%, 1.6%, and 1.8%, respectively. Compared with K-means, FLA achieves improvements of 12.6%, 6.1%, and 9.0%, respectively. These results demonstrate that FLA exhibits relatively stable performance across different datasets compared with traditional baseline methods.

In comparison with deep learning–based methods, scLTF shows consistently better clustering performance. Specifically, in terms of ARI, NMI, and ACC, scLTF consistently outperforms scMAE, scDeepCluster, and scVI+Leiden across all 8 datasets. It also outperforms scTPC on 7 out of 8 datasets, and consistently achieves better results than FLA across all datasets. In terms of average improvement, scLTF achieves gains of 13.3%, 7.7%, and 8.7% over scMAE in ARI, NMI, and ACC, respectively. Compared with scDeepCluster, the improvements are 24.1%, 9.4%, and 18.7%, respectively. Compared with scVI+Leiden, scLTF improves by 17.1%, 10.5%, and 11.5%, respectively. Compared with scTPC, the improvements are 4.4%, 3.9%, and 5.1%, respectively. Compared with FLA, scLTF achieves improvements of 10.1%, 6.2%, and 8.7%, respectively. These results indicate that scLTF consistently achieves stable performance improvements over both the initial clustering method (FLA) and other deep learning–based approaches across different datasets, indicating good cross-dataset robustness.

Second, from the perspective of computational efficiency, FLA requires less runtime than Seurat, Scanpy, and Leiden across all 8 datasets in the traditional method comparison. On average, its runtime is 45.2%, 42.6%, and 44.5% of that of Seurat, Scanpy, and Leiden, respectively. Although FLA has a longer runtime than K-means, with an average runtime 4.96 times that of K-means, the difference is not pronounced on small-scale datasets (fewer than 150,000 cells), where the runtime of FLA remains within 150 seconds. When the dataset size exceeds 300,000 cells, the runtime of FLA is approximately 1.96 times that of K-means, indicating that it maintains reasonable scalability for large-scale datasets. Overall, while FLA achieves slightly higher clustering accuracy than Seurat, Scanpy, and Leiden, it also improves computational efficiency. Compared with K-means, it achieves higher clustering accuracy.

In comparison with deep learning–based methods, scLTF requires less runtime than scMAE, scDeepCluster, scVI+Leiden, and scTPC across all 8 datasets. On average, its runtime corresponds to 6.7% of scMAE, 32.2% of scDeepCluster, 21.3% of scVI+Leiden, and 43.9% of scTPC. As the dataset size increases, this gap becomes more pronounced. When the number of cells exceeds 300,000, the runtime of scLTF is approximately 3.6% of scMAE, 7% of scDeepCluster, 6.8% of scVI+Leiden, and 26.4% of scTPC. These results indicate that scLTF achieves improved clustering accuracy compared with existing deep clustering models, while also demonstrating favorable computational efficiency.

In summary, compared with traditional methods such as Seurat, Scanpy, Leiden, and K-means, FLA achieves a well-balanced performance between clustering accuracy and computational efficiency. Building on this, the scLTF algorithm further improves clustering accuracy while maintaining relatively high computational efficiency among deep learning-based methods, suggesting its potential for large-scale single-cell data analysis..

### Stability analysis

To further evaluate the robustness of the proposed methods, we conducted stability analysis experiments. In this experiment, all methods were independently run 10 times under the same experimental settings on the GSE220195 dataset. The results were evaluated using three metrics: ARI, NMI, and ACC. For each method, the mean values were reported, and the distribution of results across repeated runs was visualized using box plots, including whiskers, quartiles, medians, and mean values, to reflect the variability and stability characteristics of different methods under multiple independent runs.

**Fig 4:**
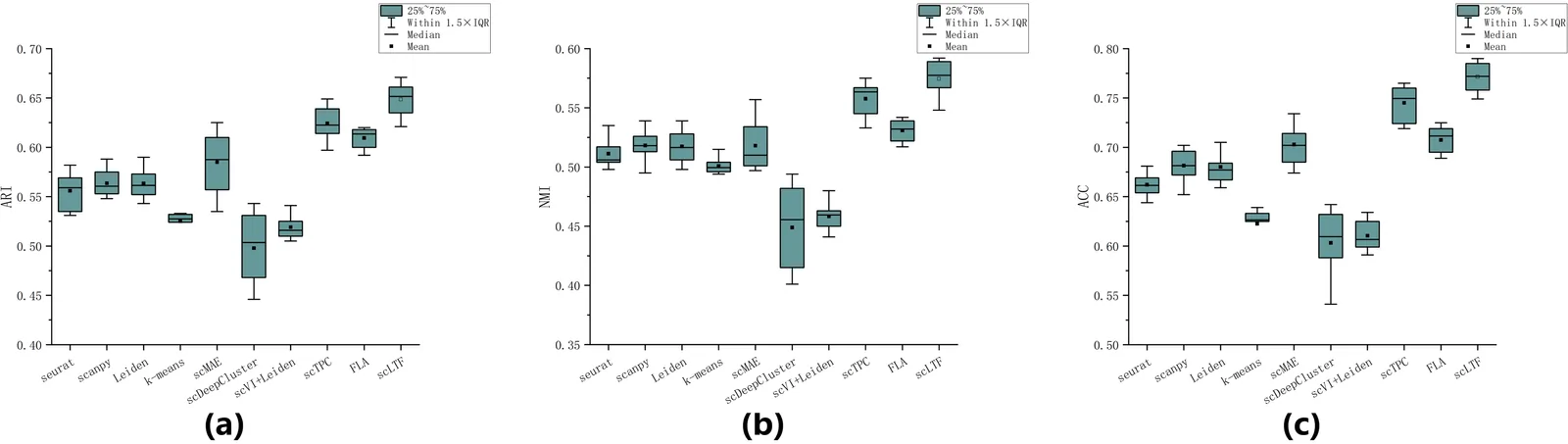
Stability analysis (box plots) of FLA, scLTF, and eight other clustering algorithms on the GSE220195 dataset. (a) ARI; (b) NMI; (c) ACC.

As shown in Fig 4, both FLA and scLTF exhibit relatively stable performance across the three metrics. Compared with Seurat, Scanpy, and Leiden, FLA shows better overall performance in terms of both stability and clustering accuracy, achieving higher values in ARI, NMI, and ACC, indicating a reasonable balance between stability and clustering performance. Compared with K-means, FLA does not show a significant improvement in stability, but it consistently achieves better performance across all three evaluation metrics.

In comparison with deep learning–based methods, scLTF also demonstrates stable performance, with a relatively concentrated distribution of results, high median values, and small interquartile ranges. It shows better robustness compared with methods such as scMAE and scDeepCluster. In addition, compared with FLA, scLTF achieves overall better performance across all three metrics, suggesting that it further improves clustering accuracy based on the initial clustering results.

Overall, the stability analysis indicates that both FLA and scLTF not only achieve good clustering performance but also exhibit limited variation across independent runs. This suggests that scLTF provides stable results for large-scale single-cell RNA sequencing data analysis, and also confirms the effectiveness of FLA as an initial clustering method.

### Visualization of clustering results

**Fig 5:**
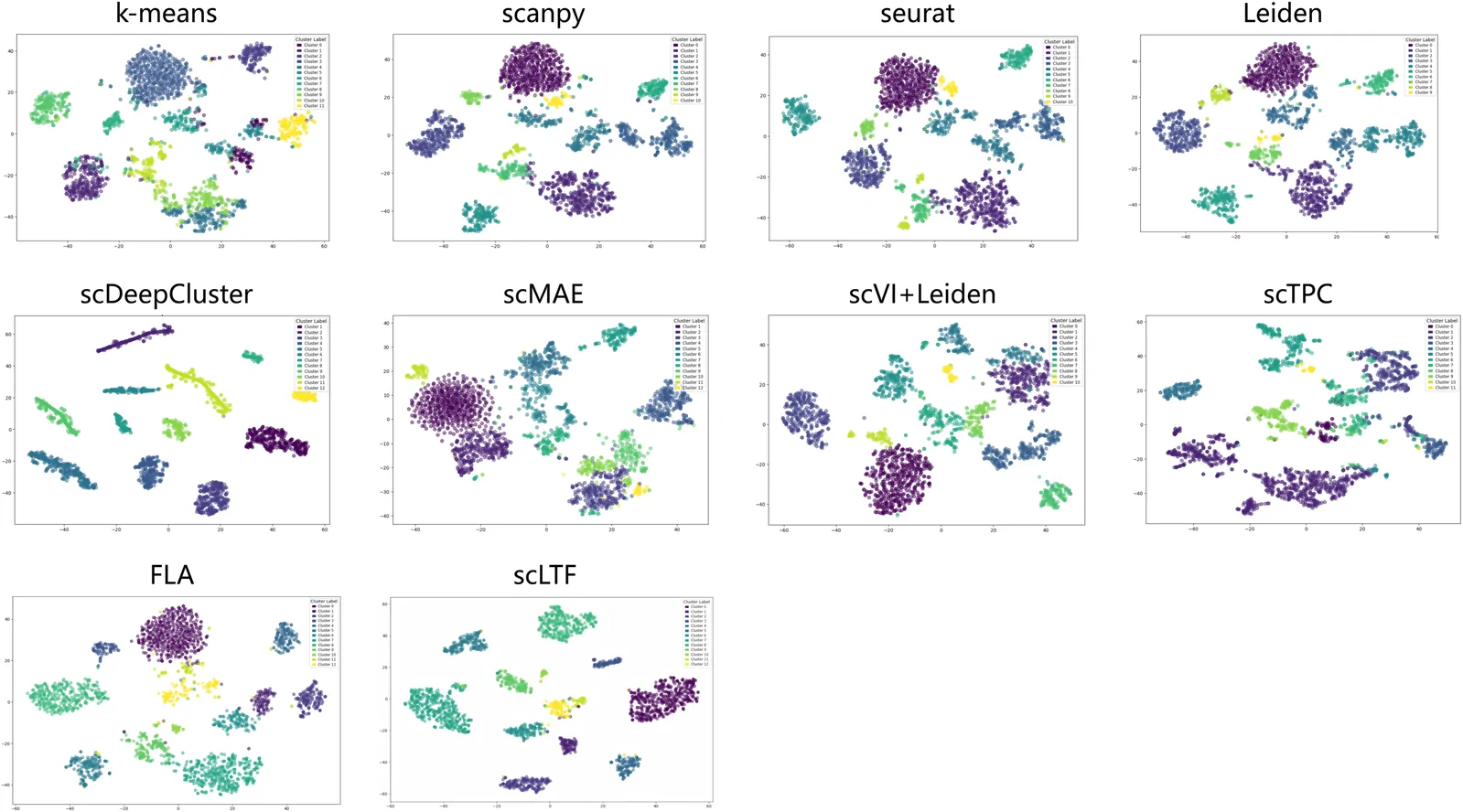
t-SNE visualization of FLA, scLTF, and eight other clustering algorithms on the GSE223414 dataset. Each point represents a single cell, and each color corresponds to a distinct cluster.

To intuitively illustrate the performance of different clustering methods on high-dimensional scRNA-seq data, we selected the GSE223414 dataset as a case study and conducted t-SNE [43] dimensionality reduction visualization analysis (as shown in Fig 5). In the figure, each point represents a cell, and different colors indicate different clusters. It should be noted that t-SNE is only used for two-dimensional embedding visualization of high-dimensional data and mainly reflects local structural relationships; therefore, this analysis serves as a qualitative supplement to the aforementioned quantitative results.

The visualization results show clear differences among methods in terms of inter-cluster separation and intra-cluster compactness. Overall, these differences are to some extent consistent with the quantitative results reported in the clustering evaluation section (ARI, NMI, and ACC), although some local discrepancies can still be observed.

Among all methods, FLA shows relatively good overall clustering structure, but some cluster boundaries are not fully distinct and intra-cluster distributions appear slightly dispersed. scLTF improves upon FLA by enhancing cluster compactness and boundary clarity, resulting in more visually coherent clustering structures. This observation is consistent with its overall superior performance in quantitative metrics, suggesting that scLTF achieves stronger intra-cluster cohesion and inter-cluster separability in the feature space. In comparison, other baseline methods show less satisfactory visualization results, with less distinct cluster structures and noticeable inter-cluster mixing.

It is worth noting that scDeepCluster exhibits relatively clear cluster separation in the t-SNE visualization; however, some clusters appear as elongated or stripe-like distributions rather than compact cluster shapes in the low-dimensional space. This suggests that the method may emphasize continuous structural representation in the embedding space rather than strictly enforcing compact cluster boundaries. Combined with the quantitative evaluation results, it can be observed that although this method shows certain separability in visualization, its visual patterns are not fully consistent with its clustering performance metrics; therefore, visualization alone should not be used to judge overall clustering quality.

Overall, the t-SNE visualization results are generally consistent with the quantitative evaluation metrics, further validating that scLTF improves clustering accuracy and preserves cellular population structure, while also visually confirming the effectiveness of FLA as an initial clustering step.

### Expression pattern visualization

**Fig 6:**
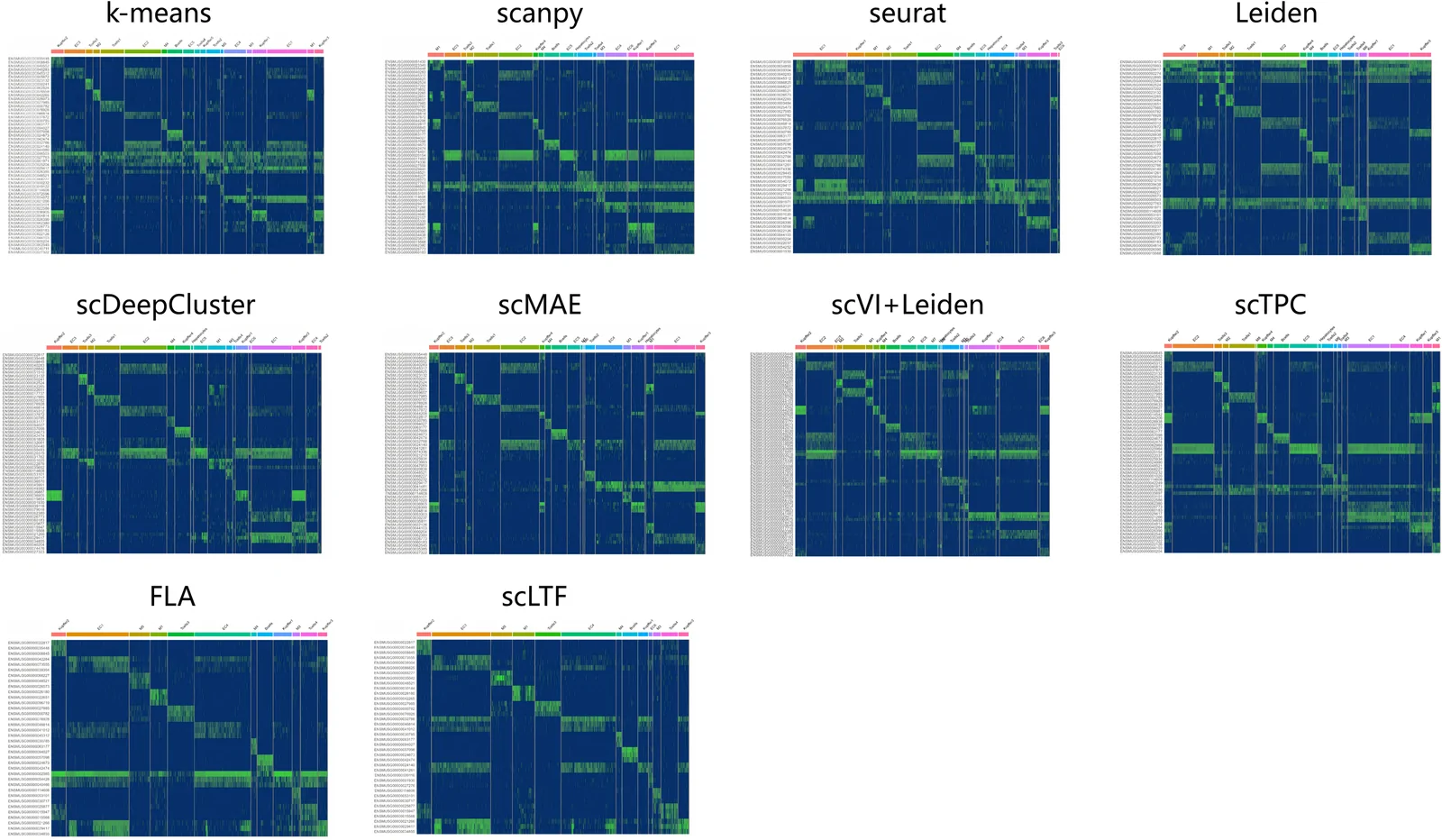
Heatmap of subpopulation marker gene expression for FLA, scLTF, and eight other clustering methods on the GSE198868 dataset.

To further assess the ability of different clustering methods to distinguish cell subpopulations at the biological level, we generated a heatmap of marker gene expression across subpopulations for the GSE198868 dataset [44] (Fig 6). Three representative marker genes were selected from each cluster to evaluate how well different clustering methods differentiate cell subpopulations. It should be noted that this analysis is primarily intended as a qualitative supplement to illustrate differences in clustering structure, rather than as an independent quantitative measure of biological validity.

The visualization results show that the four traditional clustering methods—Seurat, Scanpy, Leiden, and K-means— exhibit certain limitations in resolving subpopulation structures. In most clusters, the marker gene expression is relatively dispersed, resulting in poorly defined subpopulation features, indicating limited biological discrimination ability. The four deep learning-based methods (scDeepCluster, scMAE, scVI+Leiden, and scTPC) perform well for marker gene expression in some subpopulations, but overall cluster marker expression remains insufficiently concentrated and limited cluster separability, with partial overlap among clusters. FLA can effectively capture the marker gene distribution of each subpopulation, with most clusters showing relatively concentrated high-expression regions, though some clusters still exhibit slightly dispersed marker gene expression or overlap with other clusters. scLTF further improves upon FLA, enhancing the separation of subpopulation clusters. Marker genes are more concentrated within clusters, and potential misclassification and mixing are reduced. Overall, scLTF achieves the most favorable performance among the compared methods. In general, the expression pattern differences observed in the heatmaps are broadly consistent with the quantitative results reported in the clustering evaluation section (ARI, NMI, and ACC).

In summary, the heatmap-based analysis of marker gene distributions, as a qualitative supplement, suggests that scLTF not only improves clustering accuracy on top of the initial FLA clustering, but also enhances the robustness of identifying biologically meaningful cell subpopulations. This further demonstrates the overall effectiveness of scLTF in large-scale single-cell data analysis.

### Parameter analysis

**Fig 7:**
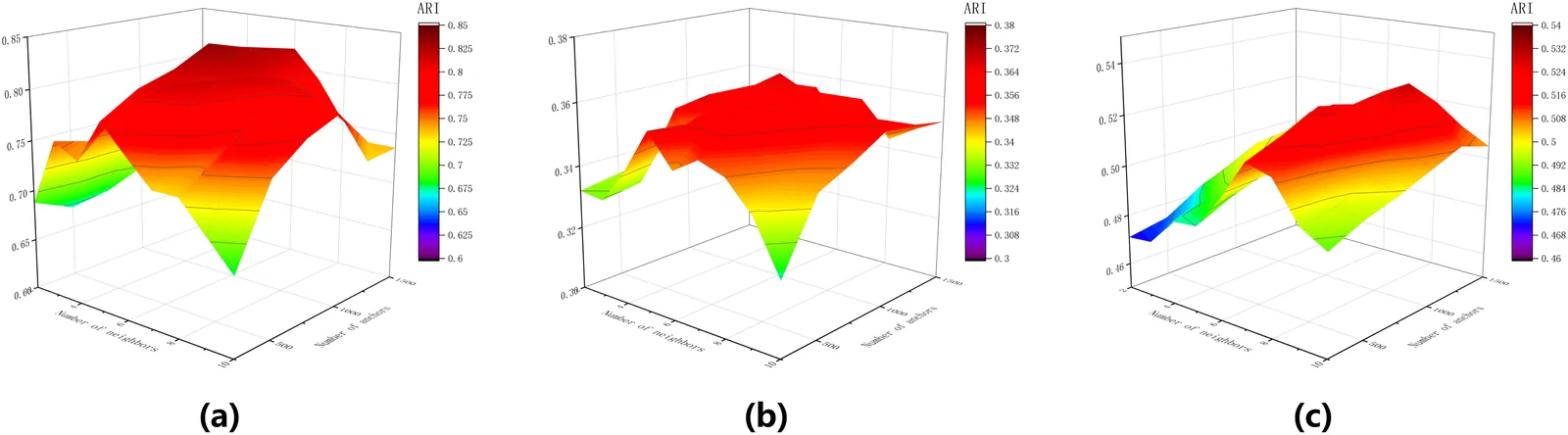
Testing results of anchor number (p) and neighbor number (k) on three datasets. (a) GSE198868; (b) 128k Mouse; (c) MCA DGE.

To systematically assess the impact of key parameters in FLA on clustering performance, we used ARI as the evaluation metric and analyzed the effects of the number of anchors (p) and the number of neighbors (k) on three datasets, namely GSE198868, 128k Mouse, and MCA DGE. Fig 7 presents the clustering results under different parameter combinations in a 3D visualization, where p was varied within the range of 250–1500 and k within 2–10. Note that the case of k = 1, which leads to extremely poor clustering performance and causes numerical scale imbalance that obscures the main trends, is not shown in the figure.

The 3D distribution results show that ARI exhibits similar trends across the three datasets. When the number of anchors is relatively small (p = 250–500), the representation of the data structure is insufficient, leading to relatively large performance fluctuations. When p increases to 750–1250, ARI reaches a generally higher level, and performance is most stable around p = 1000, indicating that anchors at this scale provide a good balance between feature coverage and redundancy control. Further increases in the number of anchors do not improve performance and may slightly decrease it, indicating that excessive anchors might introduce redundancy or noise, thereby reducing the model’s discriminative ability.

The number of neighbors also has a noticeable impact on clustering performance. With few neighbors (k = 2–3), the algorithm fails to accurately capture local adjacency, resulting in suboptimal clustering. As k increases, ARI improves and reaches relatively high values in the range of k = 5–8, suggesting that a moderate neighborhood size helps balance local and global structural information. When k becomes larger (k > 8), performance slightly decreases, possibly due to overly large neighborhoods that blur cluster boundaries and reduce inter-cluster separability.

Overall, Fig 7 clearly illustrates the effect of parameter selection on model performance. Moderate values of anchors and neighbors achieve a balance between accuracy and stability, allowing the model to maintain good clustering performance across multiple parameter settings. Results across multiple datasets further indicate that these parameter settings exhibit consistent and stable performance, providing an empirical reference that is transferable across datasets and suggesting that FLA exhibits stable performance and consistent behavior across datasets.

**Fig 8:**
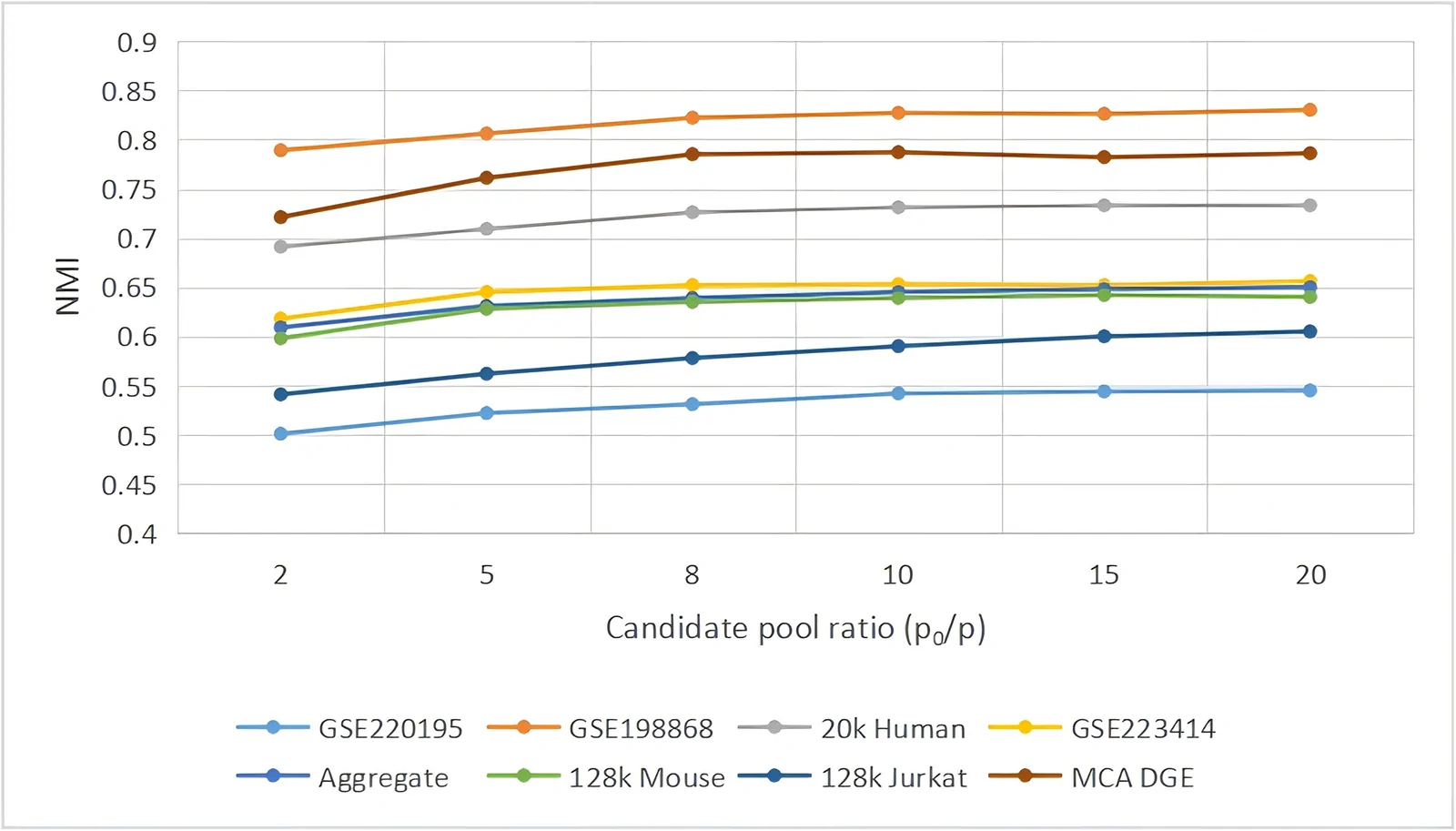
Effect of the candidate cell ratio (p_0_/p) on clustering performance across different datasets.

The number of candidate cells, p_0_, is an auxiliary parameter in the anchor generation stage. Its primary purpose is to reduce the computational cost of k-means on large-scale single-cell datasets while maintaining the representativeness of the selected anchors. To further evaluate the effect of the number of candidate cells on clustering performance, we conducted a parameter sensitivity analysis on eight real-world single-cell datasets, using NMI as the clustering performance metric. The candidate cell ratio p_0_/p was set to 2, 5, 8, 10, 15, and 20, respectively. The experimental results are shown in Fig 8.

Overall, as the candidate cell ratio p_0_/p increases, the NMI values across the eight datasets generally show a trend of initially increasing and then stabilizing, with slight fluctuations observed in some datasets at higher candidate cell ratios. When p_0_/p increases from 2 to 5, 8, and 10, the NMI values improve on most datasets, indicating that a smaller number of candidate cells may be insufficient to adequately capture the structural information of the original data, whereas increasing the number of candidate cells can improve the representativeness of the final anchors.

When p_0_/p increases to 10, the NMI values on all datasets reach relatively high levels. For example, the NMI values on the GSE220195, GSE198868, 20k Human, and 128k Jurkat datasets reach 0.542, 0.827, 0.731, and 0.590, respectively. When the ratio is further increased to 15 and 20, the changes in NMI become substantially smaller and generally stabilize, with slight decreases or fluctuations observed on some datasets. For example, on the GSE223414 dataset, the NMI changes from 0.653 (p_0_/p = 10) to 0.652 (p_0_/p = 15) and 0.656 (p_0_/p = 20); on the 128k Mouse dataset, it changes from 0.639 to 0.642 and 0.640, respectively; and on the MCA DGE dataset, it changes from 0.787 to 0.782 and 0.786, respectively. These results indicate that once p_0_/p reaches approximately 10, further increasing the number of candidate cells has a limited effect on clustering performance and does not lead to consistent performance improvements.

Overall, the final clustering performance is relatively insensitive to p_0_. A smaller candidate cell ratio may reduce the representativeness of the anchors due to insufficient coverage of the candidate cells. When the candidate cell ratio increases to approximately 10, the performance becomes largely stable, while further increasing the number of candidate cells provides limited performance gains. Therefore, considering both clustering performance and computational cost, we set p_0_ = min(10p, N) as the default setting. These experimental results provide empirical support for the choice of the number of candidate cells.

**Fig 9:**
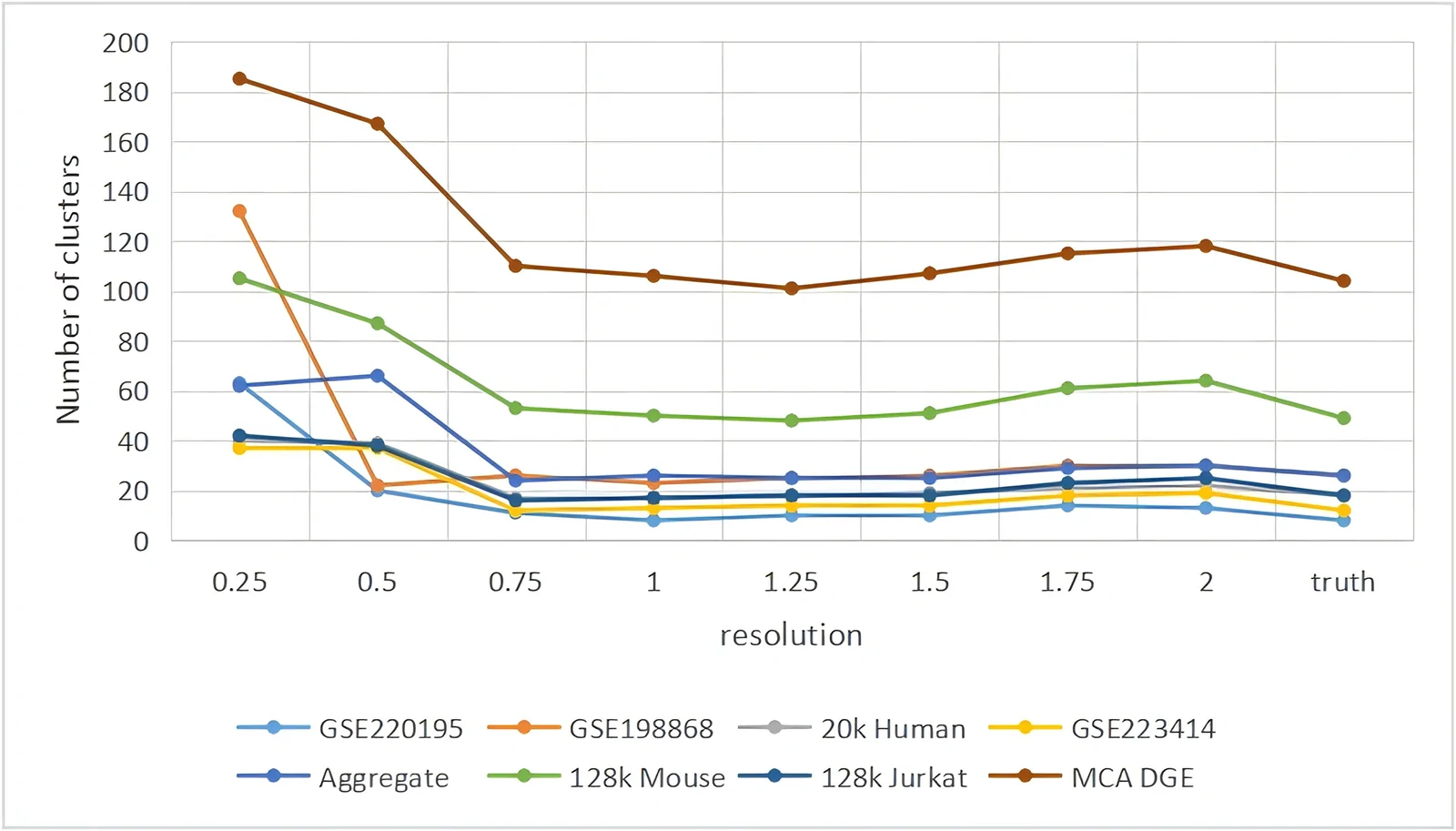
Effect of the resolution parameter on the number of clusters across different datasets.

To evaluate the impact of the resolution parameter in the FLA algorithm on clustering granularity, which directly determines the resulting number of clusters, we conducted experiments on eight real-world datasets. The resolution parameter was varied from 0.25 to 2.0, and the resulting number of clusters under different settings was recorded and compared with the true number of cell types (see Fig 9). During the experiments, all other parameters were kept unchanged to ensure that variations in clustering results were primarily attributed to the resolution parameter.

The results show that the resolution parameter has a significant effect on the number of clusters. When the resolution is low (0.25–0.5), the Louvain algorithm tends to produce excessively fragmented clustering structures, and a noticeable deviation in cluster numbers is observed in most datasets. For example, in the GSE220195 dataset, the true number of cell types is 8, whereas 63 clusters are obtained when resolution = 0.25, indicating that low resolution leads to unstable and over-segmented clustering results. A similar pattern is also observed in the GSE198868 dataset, where the true number of classes is 26, while 132 clusters are produced at resolution = 0.25.

As the resolution increases (0.75–1.25), the number of clusters gradually approaches the true number of cell types, suggesting that this range achieves a better balance between local partitioning and global consistency. For instance, in the GSE220195 dataset, when resolution = 1, the clustering result yields 8 clusters, which exactly matches the true number of cell types. In the GSE198868 dataset, when resolution = 1.25, 25 clusters are obtained, which is close to the true number of 26. Similar trends are consistently observed across other datasets.

When the resolution further increases (1.5–2.0), the number of clusters increases again, indicating a tendency toward over-partitioning. For example, in the MCA dataset, the true number of cell types is 104, while 118 clusters are obtained when resolution = 2.0, suggesting that excessively high resolution leads to further subdivision of cell populations, thereby increasing clustering complexity and reducing consistency with the underlying biological structure.

Overall, results across different datasets indicate that FLA can stably obtain clustering structures that are close to the true number of cell types when the resolution parameter is set in the range of approximately 0.75–1.25. Within this range, the algorithm maintains sufficient resolution for distinguishing subpopulations while avoiding excessive splitting or merging. These findings suggest that FLA is relatively robust to the resolution parameter in the Louvain clustering process and can reliably recover biologically meaningful clustering structures across datasets, providing an empirical reference for parameter selection in practical applications.

**Fig 10:**
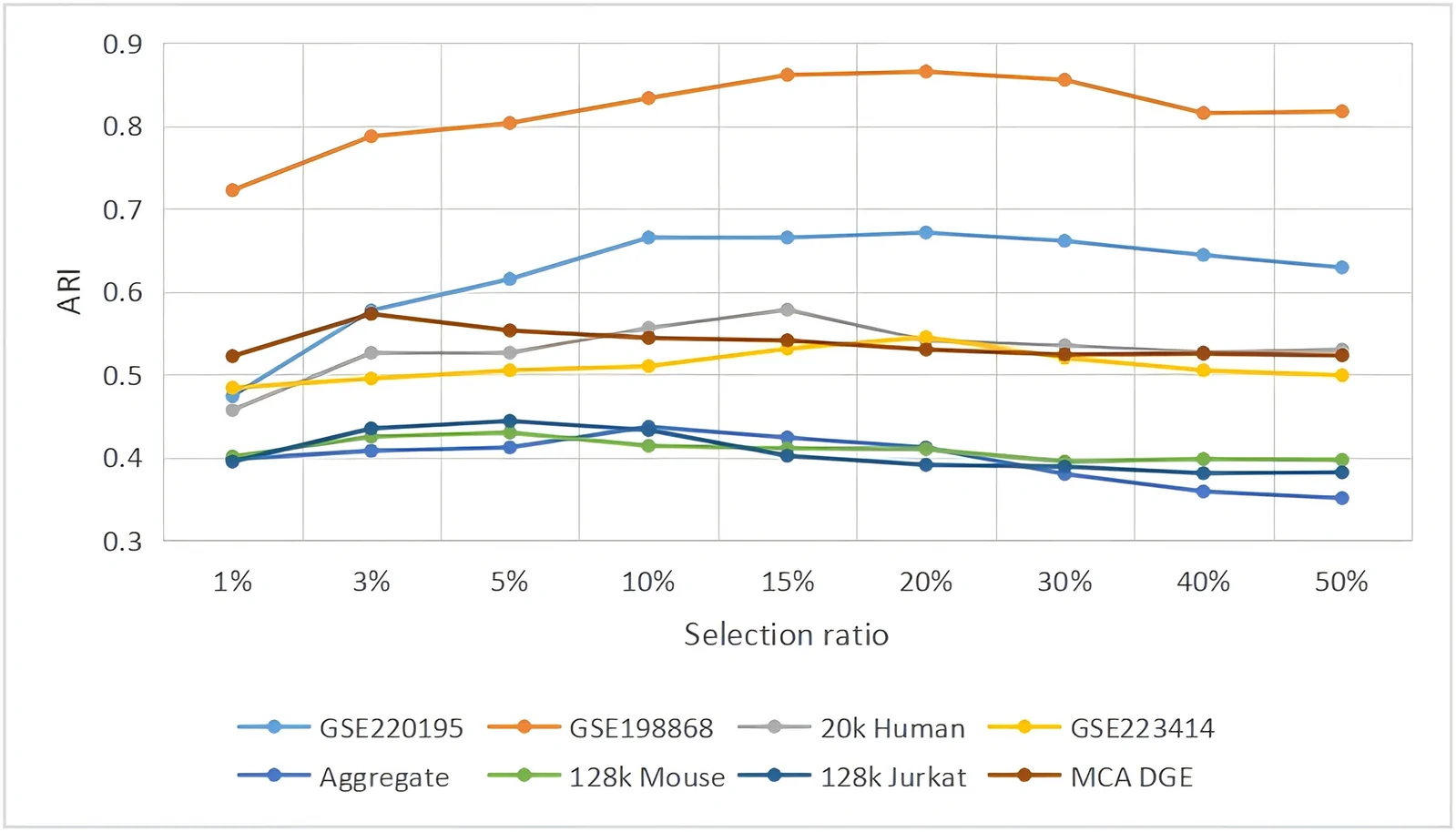
Effect of representative cell proportion on clustering performance across different datasets.

During the training process of the Transformer model in scLTF, representative cells selected based on the CRI metric have a significant impact on model performance. To investigate the effect of different proportions of representative cells on clustering accuracy, we conducted experiments on eight real single-cell datasets, varying the proportion of representative cells per cluster from 1% to 50%, and used ARI as the evaluation metric for clustering performance (see Fig 10). In the experiments, this range was used to balance information representation capacity and computational cost, while all other parameters were kept constant to ensure that variations in ARI were primarily attributed to the proportion of representative cells.

The results show that ARI exhibits a clear nonlinear trend with respect to the proportion of representative cells. When the proportion is low (1%–3%), the number of representative cells is limited, leading to insufficient intra-cluster structural representation and relatively low clustering performance. For example, in the GSE220195 and 20k Human datasets, ARI increases from 0.474/0.457 at 1% to 0.577/0.526 at 3%, indicating that a moderate increase in representative cells can effectively improve model representation capability.

As the proportion further increases (5%–20%), ARI reaches relatively high levels in most datasets and achieves peak values in some cases. For instance, in the GSE198868 dataset, ARI reaches its highest value of 0.865 at 20%; in the 20k Human dataset, the best performance (0.578) is observed at 15%; and in the Aggregate dataset, the best result (0.437) occurs at 10%. These results indicate that an appropriate proportion of representative cells can achieve a balance between information preservation and redundancy control.

When the proportion further increases to 30%–50%, ARI shows an overall decreasing trend and gradually approaches the performance of the initial clustering results from FLA. For example, in the GSE198868 dataset, ARI decreases from 0.865 at 20% to 0.817 at 50%; in the 128k Mouse dataset, ARI decreases from 0.414 at 10% to 0.397 at 50%. This suggests that excessively high proportions of representative cells do not further improve representation ability and instead introduce additional computational redundancy.

Across different datasets, a relatively consistent optimal range can be observed. For small- to medium-scale datasets (e.g., GSE220195, GSE198868, and 20k Human), better performance is achieved in the range of 10%–20%, while for large-scale datasets (e.g., 128k Mouse and MCA DGE), satisfactory performance can already be obtained within 5%–15%. This pattern indicates that smaller datasets require more representative cells to adequately capture intra-cluster structure, whereas larger datasets can achieve stable representation with lower proportions.

Overall, by appropriately adjusting the proportion of representative cells, the model can effectively control redundancy while preserving intra-cluster structural information, thereby improving both Transformer training efficiency and clustering performance. The results demonstrate that a relatively stable and appropriate range exists across datasets of different scales, providing an empirical reference for the CRI-based representative cell selection strategy in scLTF.

**Fig 11:**
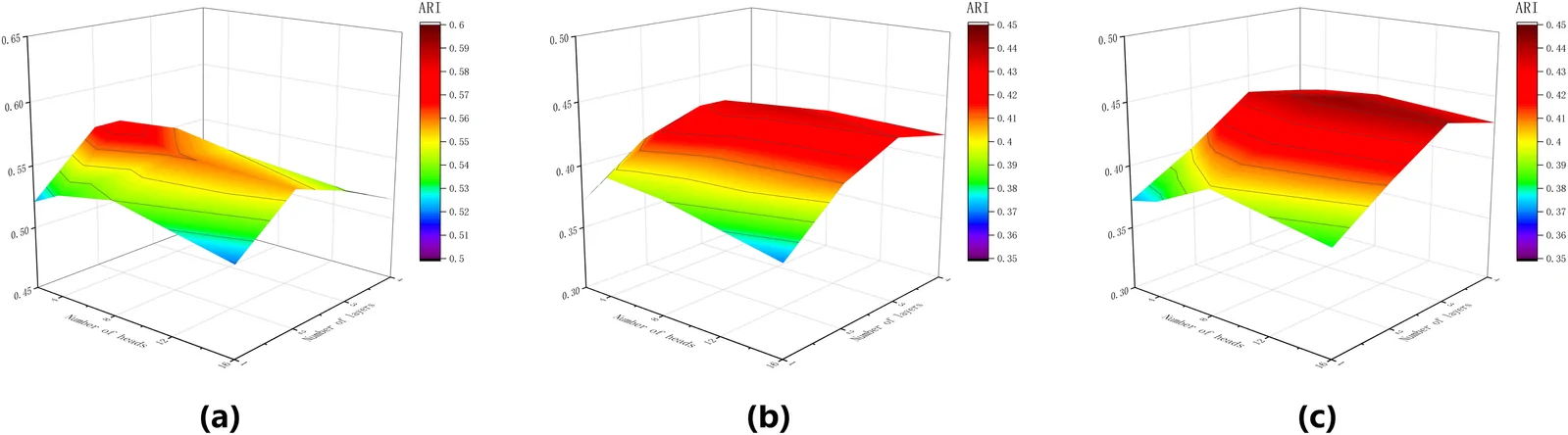
Evaluation results of encoding layer number and attention head number on three datasets. (a) 20k Human; (b) Aggregate; (c) 128k Jurkat.

In addition to the influence of representative cell proportion on clustering performance, the internal structural parameters of the Transformer model also affect the final clustering results. Therefore, we further conducted a systematic evaluation of combinations of the number of encoding layers and the number of attention heads on three datasets, namely 20k Human, Aggregate, and 128k Jurkat. Fig 11 presents the clustering performance (ARI) under different parameter settings.

The results indicate that the Transformer model shows a certain degree of sensitivity to the number of encoding layers and attention heads; however, the overall impact is relatively limited, and performance differences across different parameter combinations are relatively smooth. When the number of encoding layers is small (one layer), the model’s representational capacity is constrained, resulting in generally lower ARI across datasets. As the number of encoding layers increases to 2–3, clustering performance improves and reaches a relatively good range. For example, in the 20k Human dataset, the best performance is achieved with 2 encoding layers and 4 attention heads (ARI = 0.578); in the Aggregate dataset, the optimal result is obtained with 3 encoding layers and 8 attention heads (ARI = 0.437); and in the 128k Jurkat dataset, the best performance is also achieved with 3 encoding layers and 8 attention heads (ARI = 0.444).

The effect of the number of attention heads is relatively weaker and shows mild fluctuations. When the number of heads is small (two heads), the ability to model multi-scale features is limited, resulting in slightly lower performance. As the number of attention heads increases to 4–8, performance improves across datasets and reaches a relatively favorable range. When the number of heads further increases to 16, no significant improvement is observed, and in some cases performance slightly decreases, suggesting that an excessive number of attention heads may reduce the representational capacity of each head, thereby affecting the overall quality of representation.

Overall, there is a certain coupling relationship between the number of encoding layers and the number of attention heads. A combination of moderate depth (2–3 layers) and a moderate number of attention heads (4–8 heads) achieves a good balance between representational capacity and feature decomposition ability, leading to stable and relatively strong performance across datasets.

In summary, results across the three datasets indicate that the Transformer structural parameters exhibit a general pattern of “prioritizing stability with relatively low sensitivity.” Insufficient encoding depth limits representational capacity to some extent, while further increases bring only marginal benefits. The number of attention heads has a relatively minor effect and mainly provides fine-grained adjustment. These findings suggest that the Transformer module in scLTF is relatively robust to parameter settings, providing a stable empirical reference for practical applications.

### Cell–Anchor label consistency analysis

To address the possibility that a cell and its nearest anchor may be assigned to different communities during the Louvain community detection process in FLA, we further perform a quantitative analysis of cell–anchor label consistency. For each cell, its corresponding nearest anchor is determined during the local neighboring anchor selection process described above. Based on this assignment, we further compare whether the community labels of the cell and its nearest anchor are consistent. If they have the same community label, the cell and its nearest anchor are considered label-consistent, and the cell–anchor label consistency rate is calculated accordingly.

The experimental results are presented in Table 3. The cell–anchor label consistency rates across the eight real-world scRNA-seq datasets all exceed 93%, with the lowest rate observed on the GSE198868 dataset (93.29%) and the highest on the 128k Jurkat dataset (97.89%), yielding an average consistency rate of 95.55%. These results indicate that, after community detection based on the cell–anchor bipartite graph, the vast majority of cells and their corresponding anchors are assigned consistent community labels. This finding further demonstrates that, although the Louvain community detection process does not explicitly constrain each cell and its nearest anchor to belong to the same community, their community labels exhibit a high degree of consistency in the actual community detection results.

**Table 3:** Cell–anchor label consistency statistics across the eight datasets.

Dataset	Total number of cells	Number of consistent cells	Consistency rate
GSE220195	11,754	11,382	96.84%
GSE198868	23,736	22,143	93.29%
20k Human	23,837	22,525	94.50%
GSE223414	45,460	43,604	95.92%
Aggregate	66,457	64,177	96.57%
128k Mouse	113,001	106,080	93.88%
128k Jurkat	145,628	142,558	97.89%
MCA DGE	333,778	318,744	95.50%

**Fig 12:**
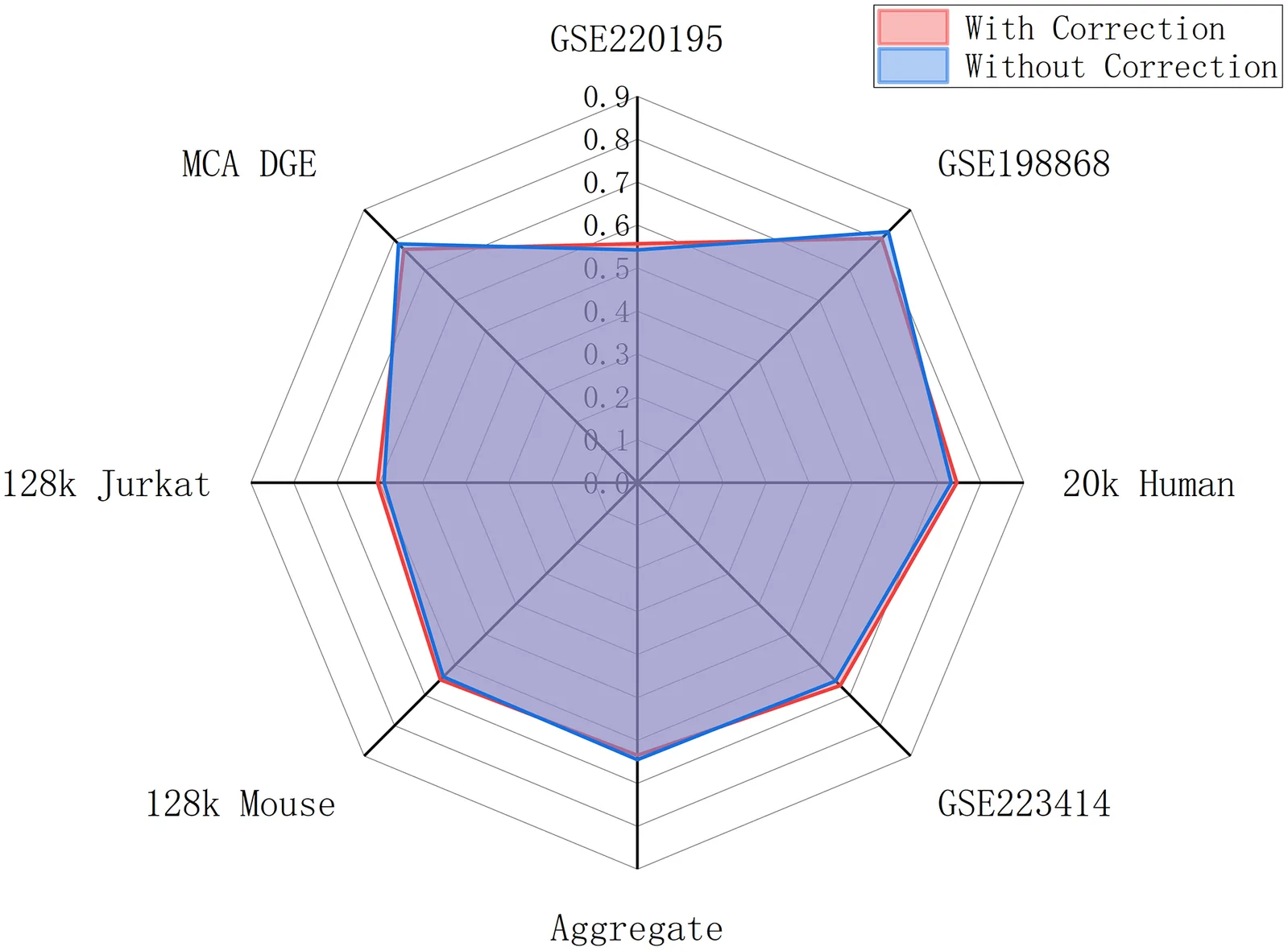
Comparison of NMI before and after cell–anchor label correction.

To further investigate whether explicitly resolving the small number of cell–anchor label inconsistencies is necessary, we conducted an additional label correction experiment. With all other experimental settings kept unchanged, the FLA cluster label of each cell was reassigned to the label of its nearest anchor, and the original FLA clustering results without label correction were used as the control. NMI was used as the clustering performance metric, and the experimental results are shown in Fig 12.

The results show that introducing cell–anchor label correction leads to only minor changes in clustering performance across the different datasets, with an average relative improvement in NMI of only 0.56% across the eight datasets. Among them, three datasets show a decrease in performance after label correction, while the performance gains on the remaining datasets are also relatively limited. These results indicate that, although label inconsistencies exist between a small number of cells and their corresponding anchors, introducing an explicit label correction mechanism does not provide consistent and substantial improvements in clustering performance across different datasets. Therefore, the existing FLA clustering strategy does not require this additional correction step.

Taken together, the consistency analysis and label correction experiments show that, although community label inconsistencies exist between a small number of cells and their corresponding anchors, the proportion of such inconsistencies is relatively low, and correcting these inconsistencies does not result in significant performance gains. Therefore, we do not introduce an additional cell–anchor label correction mechanism into FLA. The community labels assigned to cells by the Louvain algorithm on the overall bipartite graph are retained as the preliminary clustering results.

### Ablation study

**Fig 13:**
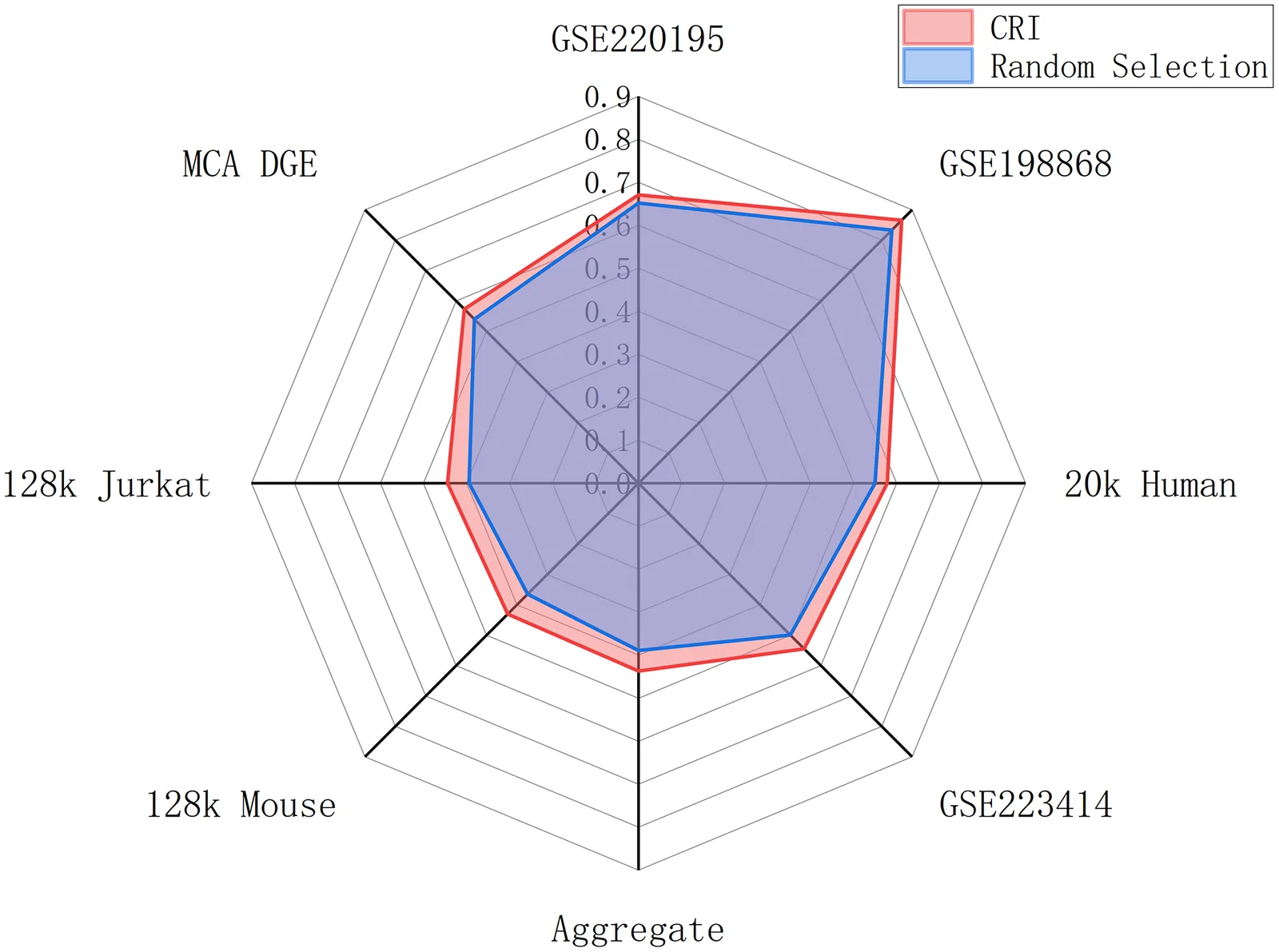
Comparison of ARI performance between CRI and random selection across eight datasets.

To evaluate the effectiveness of the Clustering Representativeness Index (CRI) in selecting representative cells, we compared cells chosen based on CRI with randomly sampled cells from each initial cluster, using ARI as the metric for clustering performance. Fig 13 presents a comparison of ARI performance between the two strategies across all datasets. The results indicate that cells selected via CRI consistently outperform the random selection strategy, with an average improvement of approximately 8.8%. The largest gains are observed on the 128k Mouse and 128k Jurkat datasets, with ARI improvements of 17.8% and 12.7%, respectively. This indicates that CRI more effectively captures key features in datasets with large cell numbers or complex subpopulation structures, thereby improving Transformer model training performance. Overall, this ablation study demonstrates that CRI contributes to improving the final clustering accuracy, providing empirical support for its application in single-cell clustering workflows.

**Fig 14:**
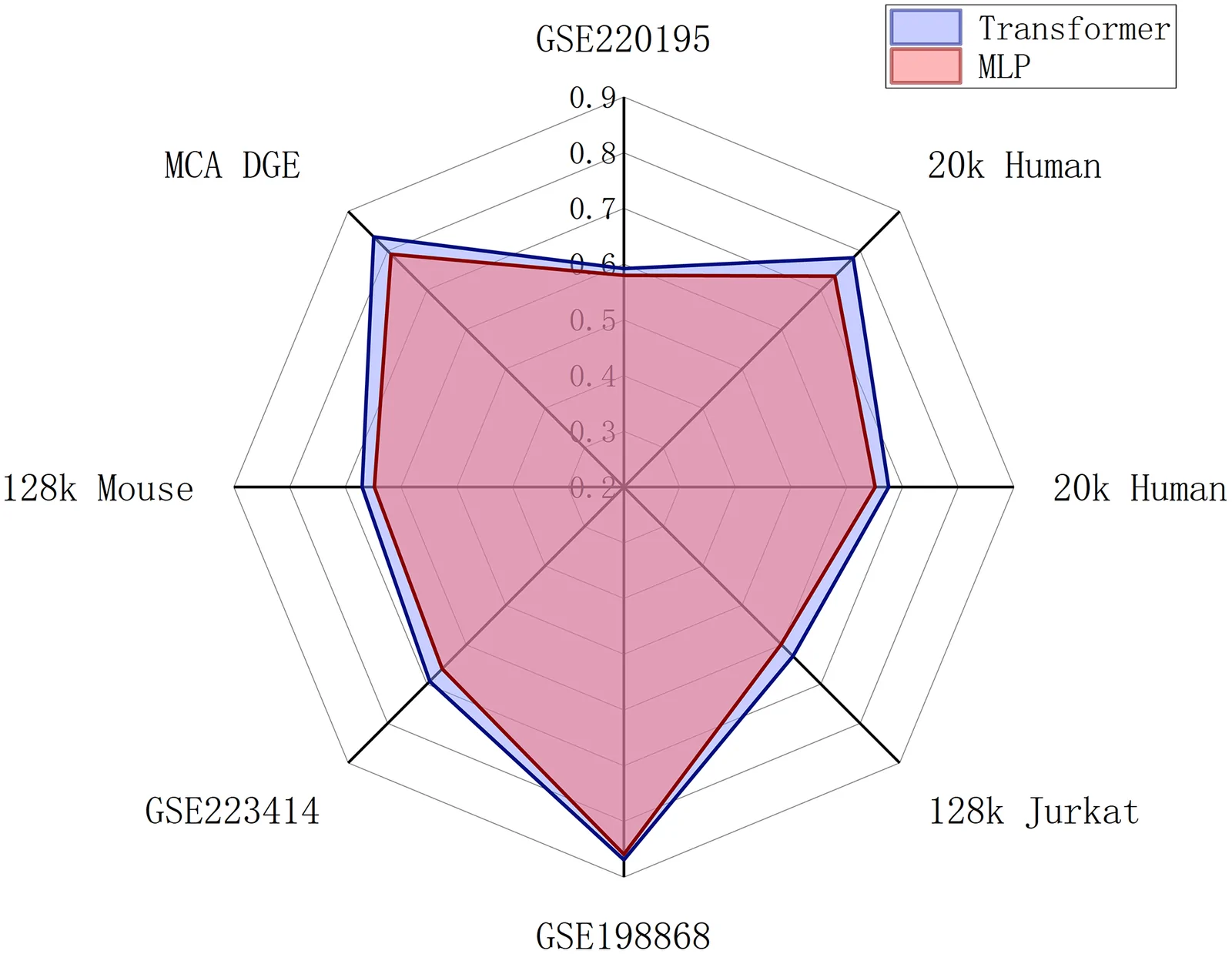
Comparison of NMI performance between Transformer and MLP across eight datasets.

To further validate the effectiveness of the Transformer architecture in the proposed method, we replaced the Transformer with a Multi-Layer Perceptron (MLP) while keeping the CRI-based cell selection strategy and all other experimental settings unchanged, and conducted comparative experiments. Normalized Mutual Information (NMI) was used as the evaluation metric for clustering performance. Fig 14 presents the performance comparison between the Transformer and MLP models across eight datasets.

The results show that the Transformer-based model consistently outperforms the MLP across all datasets, with an average improvement of approximately 4.0%. The most notable gains are observed on the 20k Human and MCA DGE datasets, where NMI increases by 6.4% and 5.7%, respectively.

Overall, the Transformer model demonstrates superior clustering performance across diverse scRNA-seq datasets. These results indicate that, compared with the MLP, which relies primarily on fully connected layers for feature mapping, the Transformer is more effective in modeling complex nonlinear feature relationships, thereby contributing to improved clustering performance.

## Conclusion

This study addresses the performance bottlenecks of traditional clustering methods in high-dimensional, noisy, and large-scale single-cell RNA sequencing (scRNA-seq) data by proposing a clustering framework, scLTF, to improve clustering efficiency, accuracy, and stability. The proposed method first performs preliminary clustering using a fast Louvain algorithm (FLA), then introduces a Clustering Representativeness Index (CRI) to select informative cells, and further employs a Transformer model for deep feature extraction and clustering refinement.

Experimental results show that scLTF achieves competitive or superior performance compared with multiple baseline methods across several real scRNA-seq datasets, with consistent improvements in evaluation metrics including ARI, NMI, and ACC. Compared with deep learning-based approaches, scLTF maintains competitive clustering performance while offering higher computational efficiency, demonstrating its potential suitability for large-scale data analysis. In addition, the method shows stable performance across datasets with varying sizes and noise levels, and exhibits good scalability on large-scale datasets.

Although scLTF shows good performance in large-scale single-cell data analysis, its performance is somewhat dependent on the settings of initial clustering parameters and the strategy for selecting representative cells. Future work will focus on developing adaptive parameter selection, improving cross-dataset generalization, and exploring integration with multi-omics data to further enhance robustness and interpretability. Moreover, owing to its efficiency and stability, scLTF may have potential applications in biomedical research, including disease subtyping, cell state identification, and precision medicine.

## Supporting information

S1 File Experimental environment and settings. (DOCX)
