# Supplementary material for: scLTF: A self-training clustering framework for large-scale single-cell RNA-Seq data
Source: PLoS One. 2026 Sep 24;21(9):e0359153. doi: 10.1371/journal.pone.0359153 (PMC13606938; doi:10.1371/journal.pone.0359153)
Supplement: Supplementary file 1 [file pone.0359153.s001.docx]

To ensure fair comparisons among different methods and the reproducibility of the results, all competing methods were evaluated under a unified experimental environment. All experiments were conducted on the same computational platform, with the hardware and software configurations described belowl.

## Hardware configuration

CPU: 2 × Intel Xeon Gold 6330 @ 2.00 GHz

GPU: 2 × NVIDIA A800 80 GB PCIe

Memory: 512 GB DDR4

Storage: 4 TB NVMe SSD + 20 TB HDD array

## Software configuration

Operating system: Windows Server 2016

Python: 3.10

R: 4.4.1

PyTorch: 2.5.1+cu121

CUDA runtime: 12.1

Scanpy: 1.11.5

NumPy: 1.26.4

Pandas: 2.3.3

scikit-learn: 1.7.2

Conda: 23.10.0

To ensure fair comparisons of clustering accuracy and runtime across different methods, all methods were evaluated using their official implementations, publicly available code, or standard implementations reproduced according to the descriptions in the original publications. Runtime measurements were performed under the same hardware environment. For methods supporting GPU acceleration, such as scVI and scMAE, CUDA acceleration was enabled. Methods implemented for CPU execution, such as Seurat and Leiden/Louvain-based methods, were consistently run in CPU mode. For GPU-based methods, necessary warm-up runs were performed before runtime measurements to reduce fluctuations caused by initialization overhead.
